# Phylogenetic analysis of the diacylglycerol kinase family of proteins and identification of multiple highly-specific conserved inserts and deletions within the catalytic domain that are distinctive characteristics of different classes of DGK homologs

**DOI:** 10.1371/journal.pone.0182758

**Published:** 2017-08-22

**Authors:** Radhey S. Gupta, Richard M. Epand

**Affiliations:** Department of Biochemistry and Biomedical Sciences, McMaster University, Hamilton, Ontario, Canada; University of Georgia, UNITED STATES

## Abstract

Diacylglycerol kinase (DGK) family of proteins, which phosphorylates diacylglycerol into phosphatidic acid, play important role in controlling diverse cellular processes in eukaryotic organisms. Most vertebrate species contain 10 different DGK isozymes, which are grouped into 5 different classes based on the presence or absence of specific functional domains. However, the relationships among different DGK isozymes or how they have evolved from a common ancestor is unclear. The catalytic domain constitutes the single largest sequence element within the DGK proteins that is commonly and uniquely shared by all family members, but there is limited understanding of the overall function of this domain. In this work, we have used the catalytic domain sequences to construct a phylogenetic tree for the DGK family members from representatives of the main vertebrate classes and have also examined the distributions of various DGK isozymes in eukaryotic phyla. In a tree based on catalytic domain sequences, the DGK homologs belonging to different classes formed strongly supported clusters which were separated by long branches, and the different isozymes within each class also generally formed monophyletic groupings. Further, our analysis of the sequence alignments of catalytic domains has identified >10 novel sequence signatures consisting of conserved signature indels (inserts or deletions, CSIs) that are distinctive characteristics of either particular classes of DGK isozymes, or are commonly shared by members of two or more classes of DGK isozymes. The conserved indels in protein sequences are known to play important functional roles in the proteins/organisms where they are found. Thus, our identification of multiple highly specific CSIs that are distinguishing characteristics of different classes of DGK homologs points to the existence of important differences in the catalytic domain function among the DGK isozymes. The identified CSIs in conjunction with the results of blast searches on species distribution of DGK isozymes also provide useful insights into the evolutionary relationships among the DGK family of proteins.

## Introduction

Diacylglycerol (DAG) and phosphatidic acid (PA) are two main signalling molecules within eukaryotic cells which, through their interactions with different effector proteins, play central roles in regulating diverse cellular processes [1–6]. The main source of DAG in cells is via the hydrolysis of phosphoinositides by the enzyme phospholipase C in response to a variety of extracellular stimuli including growth factors and hormones. The DAG produced serves as a substrate for the enzyme DAG kinase (DGK), which phosphorylates it into PA. Due to the ability of DGK to convert one important signalling molecule (DAG) into another (PA), the activity of DGK in different cells is tightly controlled for maintenance of normal physiological conditions [1–6]. Because DGK plays an important role in controlling diverse cellular processes including development, cell division and proliferation, neuronal and immune responses, vascular traffic, apoptosis, cytoskeletal reorganization, etc. multiples forms of DGK are generally found in most eukaryotic organisms [1–10].

**Fig 1:**
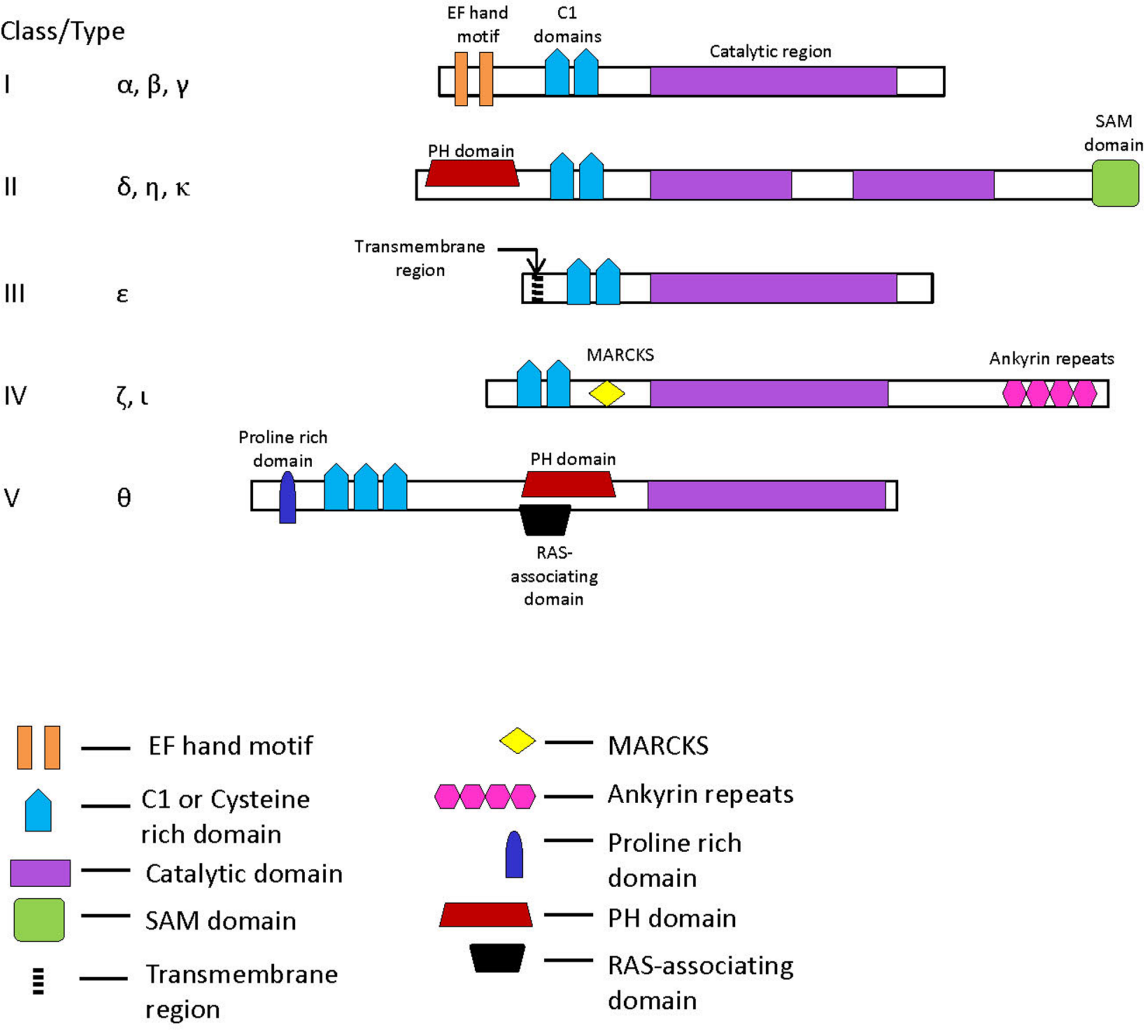
Sequence characteristics of different forms of DGK isozymes that are found in mammalian/vertebrate species. The ten known isozymes are grouped into five Classes (or Types) and a schematic of the known main functional domains in these isozymes is shown. Abbreviations; PH, plecstrin homology; MARCKS, sequence similar to the MARCKS phosphorylation site; SAM, sterile α motif. The information for the presence of different domains in the DGK isozymesis based on the following reviews [1–6,11,12].

In mammalian species, where the DGK family of proteins has been best studied, 10 different isozymes of DGK, designated as α, β, γ, δ, ε, ζ, η, θ, ι and κ, differing in their biochemical properties, tissue distributions, as well as their lengths (ranging from 567 aa to >1150 aa) have been identified [1–6,11]. Based on sequence similarities between these isozymes and the presence or absence of specific functional domains, the known DGK members have been grouped into 5 different classes or Types (Fig 1) [1–6,11,12]. All of these isozymes share in common a large catalytic domain, which is sometimes divided into two parts–catalytic and accessory domains, and 2 or 3 cysteine-rich domains, referred to as the C1 domains [reviewed in[1–6,12,13]]. The simplest and shortest (567 aa) of these isozymes is DGK-ε, sole member of the class III DGK, which contains only the commonly shared catalytic domain and the two C1 domains. In addition, DGK-ε also contains a conserved helical segment near its N-terminal end that is indicated to play an important role in its membrane interaction [12,13]. The class I DGKs (α, β and γ isozymes), in addition to containing the commonly shared domains, are characterized by the presence of two EF-hand motifs and a conserved domain of unknown function near the N-terminal end [1–6,11,12]. Novel characteristics of the class II DGK isozymes (δ, η and κ isoforms) include the presence of a plecstrin homology (PH) domain, a sterile α motif (SAM) domain and a large insert within the catalytic domain separating it into two parts. The class IV DGKs (ζ and ι isoforms) are distinguished from the others due to their containing a sequence homologous to the MARCKS phosphorylation site domain and four ankyrin repeats near the C-terminal end. Lastly, DGK-θ is the sole member of the class V DGK and it contains three C1 domains, a Gly-Pro rich domain and a PH-domain-like region with overlapping RAS-associating domain [1–6,11]. The unique domains present in DGK isozymes, through their interactions with various regulatory proteins and other molecules present in different cells and tissues, play key roles in the diverse physiological functions exhibited by different DGK isozymes [1–6].

Although the differences in the structural characteristics of DGK isozymes and their involvement in diverse cellular functions are widely known [1–6], there is limited (or no) information available at present as to how different DGK isozymes, or the different classes of DGKs, are related to each other, and have possibly evolved from a common ancestor. There is also limited understanding of the distributions of DGK isozymes in eukaryotic organisms other than mammals [3,10,14–16]. Knowledge of the species distribution and evolutionary relationships among DGK isozymes can provide useful insights into their physiological functions. Within the DGK family of proteins, the catalytic domain constitutes the single largest sequence element that is commonly and uniquely shared by all of the family members [1–6,11]. In the present work, we have used the catalytic domain sequences to construct a phylogenetic tree for the DGK family members. Additionally, and more importantly, our analysis of the sequence alignment of the catalytic domain has identified large numbers of novel sequence signatures in the forms of conserved inserts and deletions (indels) that are distinctive of either particular classes of DGK isozymes, or are commonly shared by members of two or more classes of DGK isozymes. These results point to the existence of important differences in the function of catalytic domain for different classes of DGK isozymes and are also useful in understanding the evolutionary relationships among the DGK family of proteins.

## Methods

### Phylogenetic tree construction and identification of conserved indels in the catalytic domain sequences

The protein sequences of all ten DGK isozymes from representatives of the main vertebrate groups were retrieved from the NCBI nr database. In most cases, sequences were obtained from the following species covering the diversity of the vertebrate phylum: Mammals (*Homo sapiens*, *Rattus novergicus*), Birds (*Serinus canaria*, *Sturnus vulgaris*), Reptiles (*Protobothrops mucrosquamatus*, *Python bivittatus*), Amphibia (*Xenopus tropicalis*) and Fishes (*Pundamilia nyererei*, *Maylandia zebra*). The information for accession numbers and sequence lengths of different DGK homologs that were used for phylogenetic studies is provided in the S1 Table. Multiple sequence alignments of different DGK isozymes were created using the Clustal X ver. 2.0 program [17], and based on these alignments, sequences corresponding to the commonly shared catalytic domain were identified. The large insert present within the catalytic domain (CD) of Type II DGK homologs was excluded from these alignments. The resulting sequences for the DGK CDs were then realigned and, based on these alignments, a segment of 364 aa was identified that showed good conservation amongst different DGK classes. This sequence alignment, which was used for phylogenetic analysis, is provided in S1 Fig. All of the columns with sequence gaps were not considered during tree construction leaving a total of 260 aligned positions that were used for phylogenetic analysis. The evolutionary history based on this sequence alignment was inferred using the Maximum likelihood (ML) method, with 100 bootstrap replication, using the MEGA 6 [18] based on the Jones–Taylor–Thornton (JTT) model [19]. The tree with the highest log likelihood (-7845.5131) is shown. The numbers shown on the branches identifies clades in which the associated taxa clustered together >50% time in bootstrap replicates.

Sequence alignments of the DGK homologs were also examined for the presence of conserved indels (insertions or deletions) which were flanked on both sides by at least 5–6 conserved residues in the neighboring 30–40 amino acids and which appeared to be specifically present in either a particular class of DGK isozymes, or which were shared by the DGK homologs from more one than classes [20–22]. For conserved indels meeting these criteria, additional Blastp searches were carried out using the NCBI NR (non-redundant) database to determine the specificity of the indels for the DGK classes of isozymes. These blast results were examined for the presence/absence of the identified CSIs in top 500 blast hits. SIG_CREATE and SIG_STYLE programs (available on Gleans.net) were used to format and create the signature CSIs files shown here [22,23]. Due to space limitations, sequence information for only 1–2 representative species from each vertebrate group (viz. generally the same as those employed for phylogenetic studies for mammals, birds, fishes, reptiles and amphibians) for different DGK isozymes is shown in the alignment files. However, unless otherwise noted, all of the described CSIs are specifically present in the indicated groups/classes of DGK isozymes, in different vertebrate species, where the distribution of these CSIs was studied in detail.

The presence or absence of different DGK isozymes in members from different kingdoms and main phyla of eukaryotes was assessed by carrying out Blastp searches on sequences of different DGK isozymes from human and mouse species against annotated members of specific kingdoms or phyla present in the NCBI nr database. These searches were carried out using the entire sequences of the DGK isozymes as well as utilizing sequences fonly the catalytic domain sequence. Based on significant hits observed in these Blast searches, and to which DGK isozymes the observed hits exhibited maximal similarity, tentative inferences regarding the presence or absence of specific DGK isozymes in deeper branching eukaryotic taxa were made.

## Results

### Phylogenetic analysis of the DGK isozymes based on catalytic domain sequences

**Fig 2:**
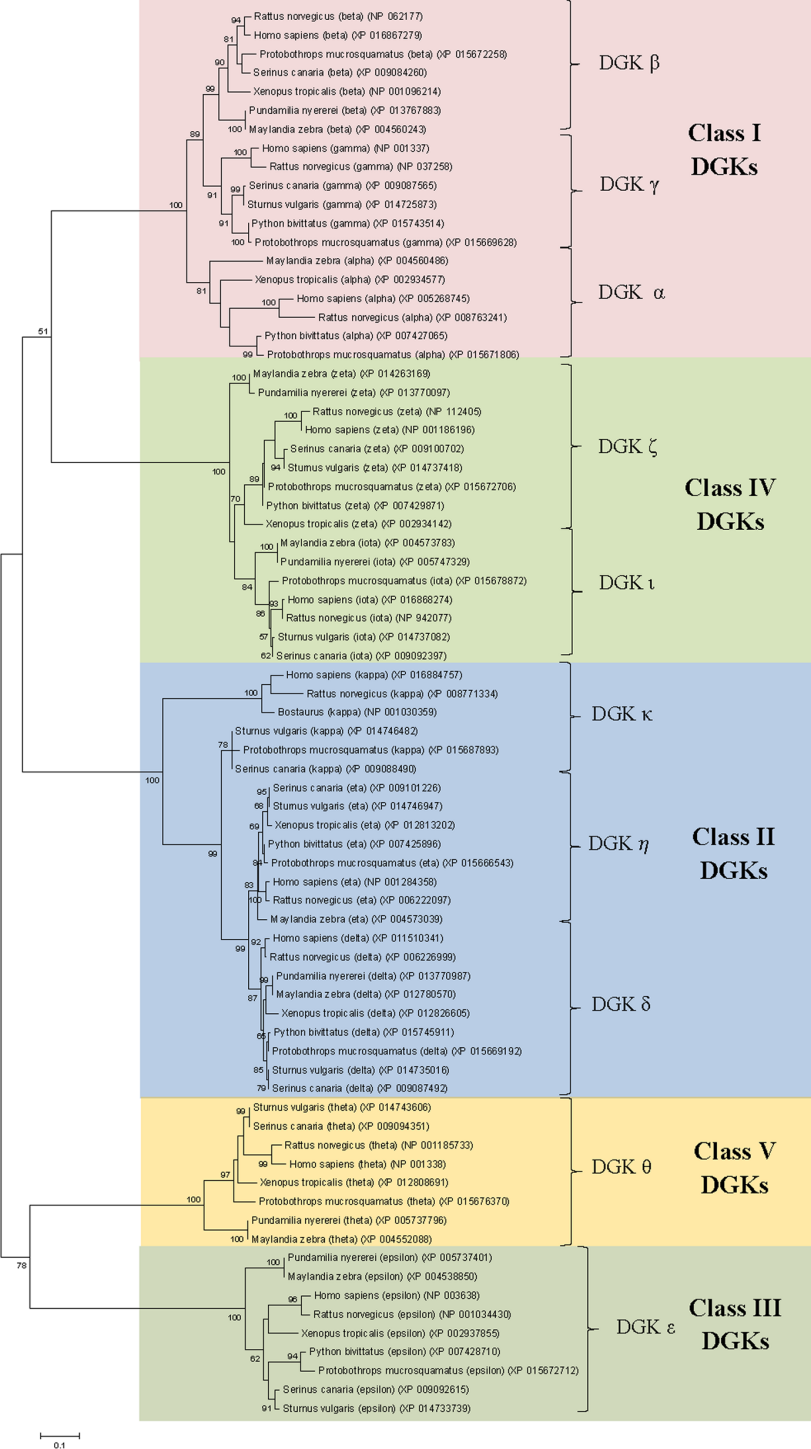
A maximum-likelihood tree for the DGK isozymes from representative vertebrate species based on the conserved regions in catalytic domain. The accession numbers of different sequences which were used for tree construction are shown after the species names. The tree was constructed as described in the Methods section and the branches which were supported by >50% bootstrap scores are marked. The tree shown was rooted at midpoint and drawn to scale, with branch lengths measured in the number of substitutions per site. Different DGK isozymes as well as the five main classes are marked.

The DGK isozymes differ considerably from each other in their sequence lengths and different domains present in them (see introduction and Fig 1). Pairwise sequence comparison studies (unpublished) indicate that the overall sequence similarity between members of different classes is in the range of 15–30% and much of the observed similarity is seen within the commonly shared catalytic domain. Based on sequence alignments of DGK homologs from representatives of different groups of vertebrates, conserved regions from the catalytic domain which could be properly aligned amongst different DGK classes were identified and these were subjected to phylogenetic analyses. A maximum-likelihood tree based on 100 bootstrap samples of this sequence region was constructed using the JTT model (see Methods) to infer the evolutionary relationships amongst different DGK homologs. The tree shown in Fig 2 is based on 260 aligned positions that were left after removal of all insertions and deletions. In this phylogenetic tree (Fig 2), DGK homologs belonging to all five classes or Types formed strongly supported clusters. These clusters were separated by long branches indicating that the DGK sequences for different classes have diverged considerably from each other even within the conserved catalytic domain. Within each DGK class, homologs belonging to different DGK isozymes also generally formed separate clusters, except for the homologs of kappa and zeta isozymes which exhibited polyphyletic branching and were separated into two clusters. The branches separating different DGK isozymes within any given class were generally short indicating that the isozymes within different classes are very similar to each other. Based on the phylogenetic tree, some information regarding relationships among different isozymes within different classes can also be gleaned. In this tree, DGK homologs belonging to the classes I and IV grouped together but there was only weak support (clustered together in 51% of bootstrap samples) for this grouping. Within the class I isozymes, the DGK-α homologs formed outgroups relative to a cluster consisting of the DGK-β and DGK-γ homologs, indicating that the sequences from the latter two groups are more closely related. Similarly, within the class II isozymes, a grouping of DGK-δ and DGK-η homologs was supported by the observed bootstrap score, with the DGK-κ sequences forming an outgroup of this cluster. Lastly, in the tree shown in Fig 2, which was rooted at midpoint, the DGK-ε and DGK-θ homologs formed the most outlying clades.

### Conserved signatures indels in the catalytic domain shared by different classes of DGK isozymes

In the tree shown in Fig 2, while the distinction between different DGK classes was strongly supported, the nodes connecting these classes had low bootstrap scores indicating that the evolutionary relationships among different DGK classes were not reliably resolved. Thus, other molecular sequence based approaches which can provide insights are needed. Conserved signature indels (CSIs) represent an important class of molecular markers that have been used in the past to resolve a number of important evolutionary questions [21,24–27]. The CSIs that are useful for evolutionary studies are generally of fixed lengths, present at specific positions iparticular genes/proteins, and they are flanked on both sides by conserved regions to ensure that they constitute reliable characteristics [21,25,27,28]. The CSIs in genes/proteins sequences generally result from rare genetic changes and the most parsimonious explanation to account for their shared presence in a given gene or protein from a specific group of species is that the genetic change giving rise to the CSI occurred in a common ancestor of the indicated group and then it was vertically inherited by the other group members [21,22,25,29]. Due to the discrete natures of the CSIs and the fact that they are located within conserved regions,their presence or absence in different lineages or proteins is generally not affected by factors such as differences in evolutionary rates among different species, or proteins, and long-branch attraction artefacts [21,24–26]. We have examined the sequence alignment of catalytic domains from DGK homologs for the presence of CSIs and these analyses have identified a number of useful CSIs that are specifically shared by particular groups of DGK isozymes.

When a conserved indel is present in a given protein, to infer whether the observed indel is an insert or a deletion, information for the presence or absence of the indel in the ancestral state of the protein is needed [22]. In most cases, such information could be obtained by determining the presence or absence of the indel in species which are ancestral to those containing the indel [25,27,28,30]. In some instances, if two proteins have evolved by an ancient gene duplication event, then the presence or absence of the indel in the other protein has also proven useful to infer whether the observed indel is an insert or a deletion [25,30]. However, due to the uniqueness of the DGK catalytic domain sequence for the eukaryotic homologs, no suitable outgroup is available for determining the ancestral state of the protein or reliably inferring whether a given CSI represents an insertion within a certain class of DGK homologs or a deletion in the rest of the DGK homologs. Hence, our use of the term insert(s) or deletion(s) to describe the identified CSIs is only in reference to the presence or absence of the same or similar sequence characteristics in other classes of DGK homologs. Additionally, it should also be acknowledged that while the presence or absence of a CSI within a given conserved region can be reliably identified (due to the sequence conservation of flanking regions), it is often difficult to specify the exact location of the CSI within a conserved region. This is especially true when the sequences of different taxa or homologs that are being compared differ considerably from each other and there is no structural information available for the proteins to guide the sequence alignments for correct placement of the CSI. Due to this limitation, the positions of various CSIs in the sequence alignment shown here represent their most likely positions based on our judgement of the sequence conservation of the flanking regions. However, we cannot exclude the possibility that in the optimal alignment that could be supported by other data the actual positions of some of these CSIs might be shifted by a few residues either to the left or on the right. Information regarding the characteristics of various CSIs that have been identified by our analysis is provided below.

**Fig 3:**
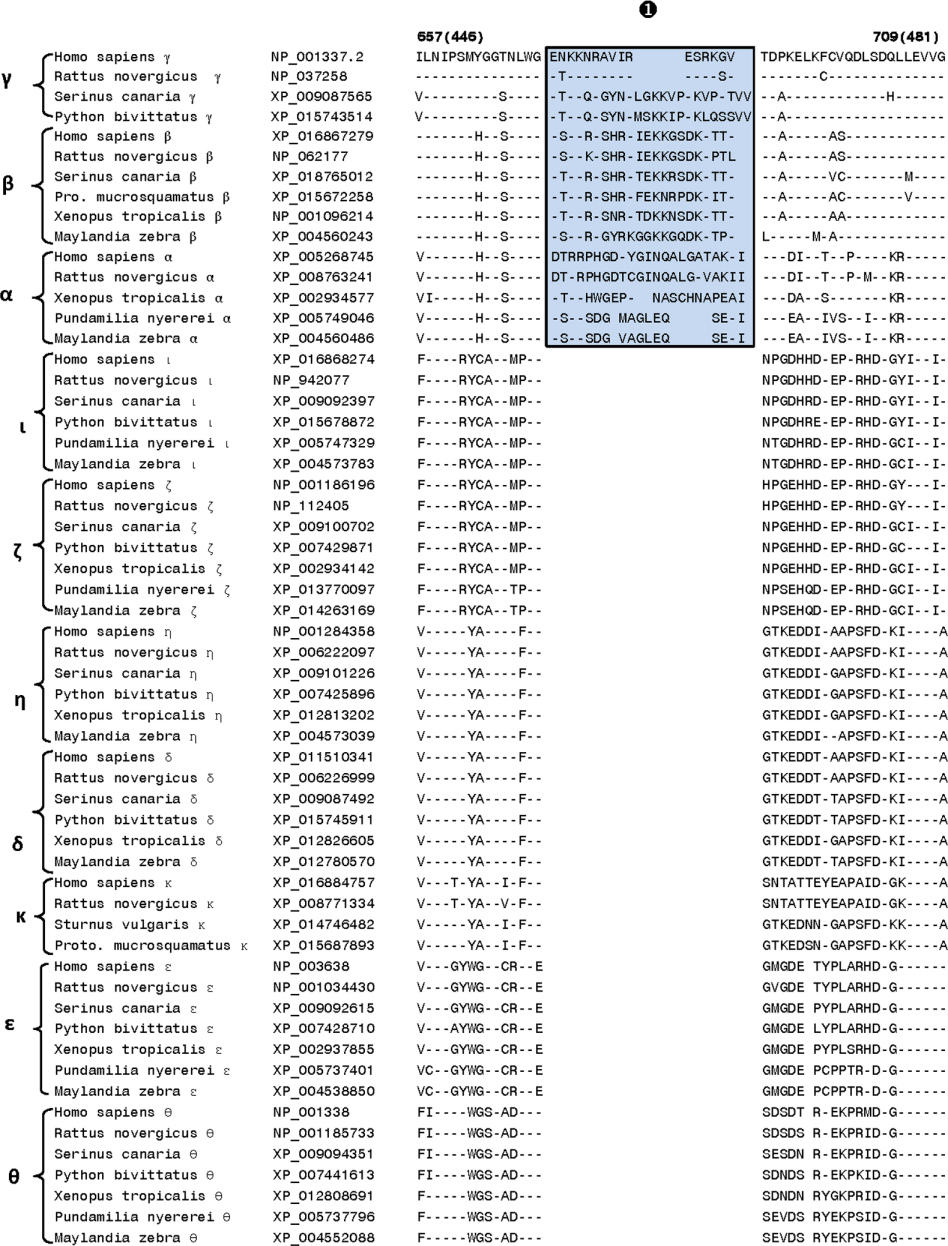
Excerpts from the sequence alignment of DGK isozymes showing a conserved insert (marked as Sig ❶) within the catalytic domain that is a unique characteristic of the Class I DGK homologs. The insert shown is present in a conserved region as the dashes (-) in the alignment denote identity with the amino acid shown on the top line. Sequence information is shown for only 1–2 representative species from different main groups within the vertebrates. However, this insert is specific for the Class I DGK homologs from all other vertebrates species examined. The second column shows the Genbank ID numbers of different sequences. The numbers on top of the alignment show the location of the depicted sequence in two reference DGK sequences. The first number outside of the parenthesis indicates the sequence position in the human DGK-γ homolog (accession number NP_001337.2) shown on the first line of the alignment, whereas the numbers within the parenthesis correspond to the location of this sequence in athe reference human DGK-ε homolog (accession number NP_003638). The vertebrate species for which the sequences are shown include the following groups: Mammals—*Homo sapiens* and *Rattus novergicus*; Birds—*Serinus canaria* and *Sturnus vulgaris*; Reptiles—*Protobothrops mucrosquamatus* and *Python bivittatus*; Amphibians—*Xenopus tropicalis* and Fish—*Pundamilia nyererei* and *Maylandia zebra*.

**Fig 4:**
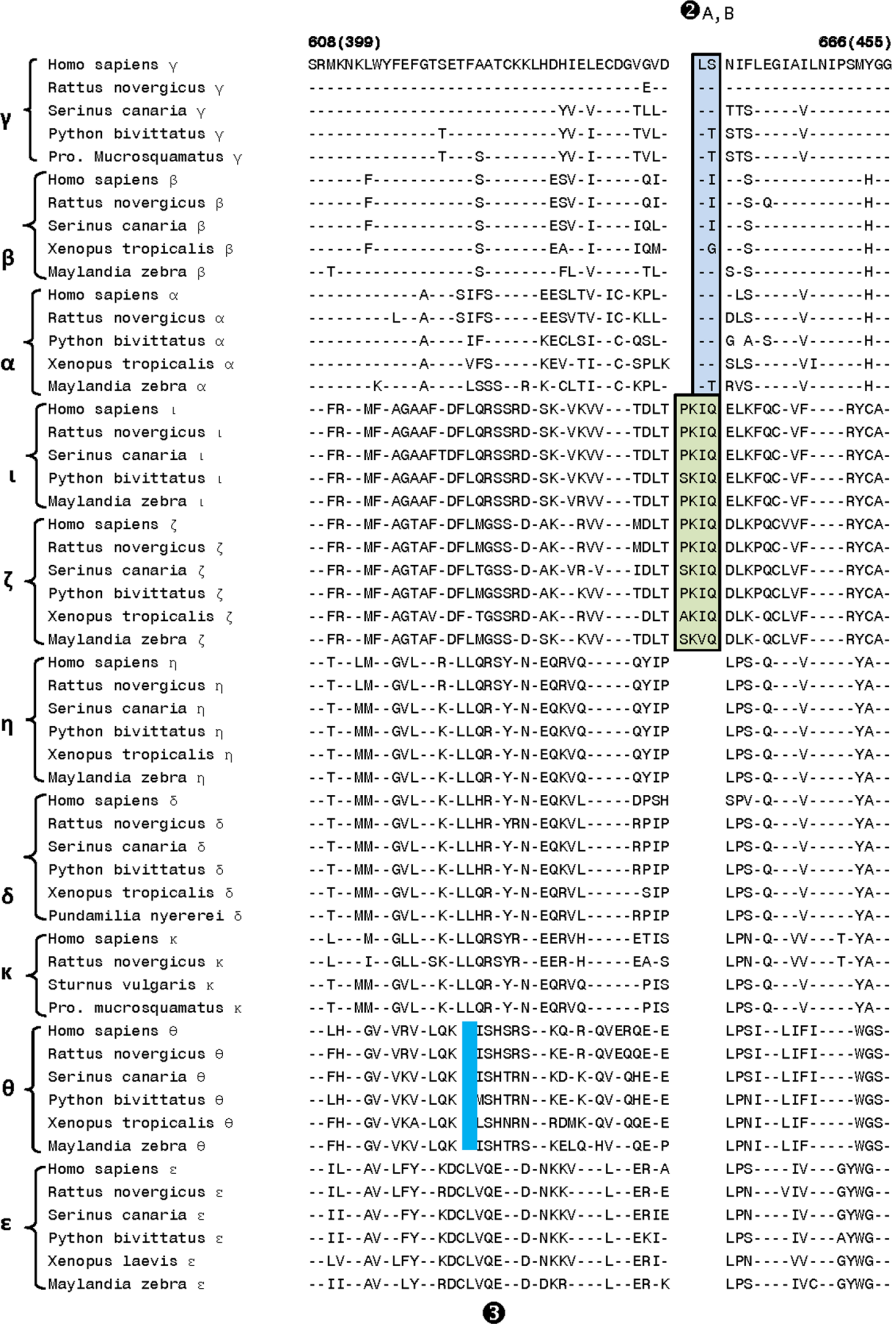
Partial sequence alignment of the DGK catalytic domain showing a number of signatures that are specific for different DGK isozymes. In the position marked Sig❷A,B, a 2 aa insert is specifically present in all class I DGK homologs, whereas in the same position a 4 aa insert is found in the class II DGK isozymes. Another signature marked ❸ consists of a 2 aa deletion that is uniquely present in the DGK-θ homologs. The signatures shown are specific for the indicated DGK isoforms in sequenced vertebrate species. The dashes (-) in the alignment denote identity with the amino acid on the top and the Genbank ID numbers for these sequences are provided in Fig 3.

The first of the identified CSIs within the catalytic domain is a 16–24 aa insert (marked signature ❶ in Fig 3) that is uniquely found in all of the Class I DGK homologs from different species. The observed CSI is present in a conserved region near the C-terminal end of the catalytic domain and its shared presence by all DGK -α, -β and -γ homologs provides further evidence of the close and specific relationship among these isozymes belonging to class I DGKs. For DGK-α and DGK-γ, interesting differences are also seen in the length of this CSI amongst the vertebrate species. For DGK-α, in contrast to the fishes (e.g. *Pundamila nyererei* and *Maylinda zebra*), homologs found in mammals (e.g. humans, *Rattus novergicus*), birds (*Serinus canaria*) and amphibians (*Xenopus tropocalis*) are 6 aa longer, suggesting that an additional insert within the DGK-α likely occurred in a common ancestor of the latter groups of vertebrate species. Similarly, for DGK-γ, the birds and reptiles harbor a longer insert in contrast to that present in mammals. Interestingly, the homologs for DGK-γ were not detected in fishes and amphibian species. In addition to the CSI shown in Fig 3, DGK homologs contain one additional 2 aa conserved insert (marked Sig ❷A in Fig 4), which is also commonly shared by the class I DGK homologs. In the same position, where this 2 aa CSI is found in class I homologs, a 4 aa insert (marked as Sig ❷B) is present in the DGK-ί and DGK-ζ homologs (class IV isozymes). These signatures serve to distinguish the Class I and Class IV DGK homologs from all others and the presence of the inserts in the same position only in these two classes of homologs suggests that they may be more closely related to each other than to the other DGK classes. In close proximity of signatures ❷A,B, another CSI consisting of 2 aa deletion (denoted by ❸ and highlighted in light blue color) is uniquely present in the DGK-θ homologs.

**Fig 5:**
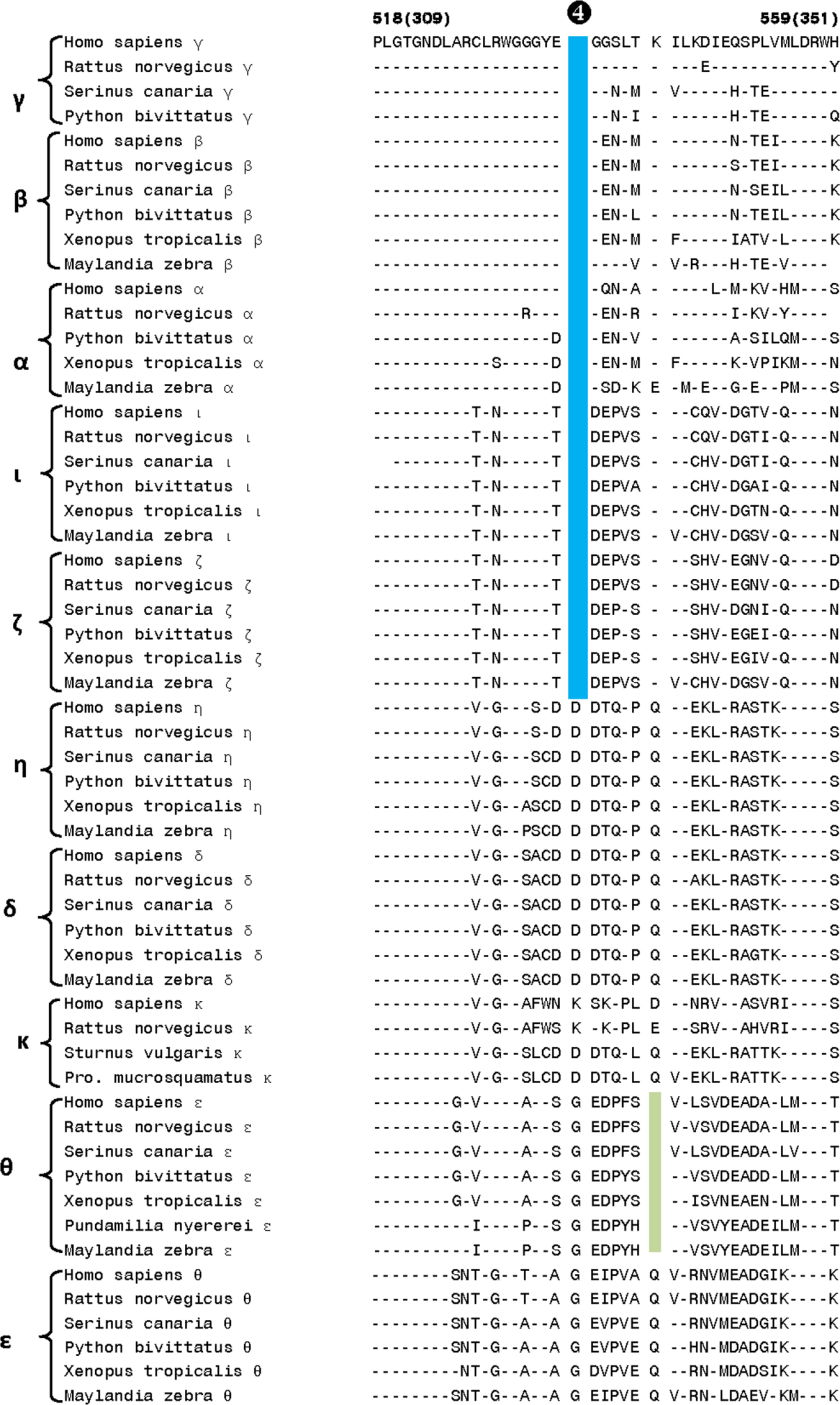
Excerpts from the multiple sequence alignment of catalytic domain from DGK isozymes showing a 1 aa conserved indel (deletion) (marked ❹) that is commonly shared by the class I and class IV isozymes. In the proximity of Sig ❹, a 1 aa deletion is also present in the DGK-θ homologs (marked as signature ❺). Although these two indels are placed in different positions, the possibility that they have occurred in the same position as ❹ cannot be excluded.

Our analysis has also identified another CSI consisting of a 1 aa deletion in a highly conserved region (denoted as Sig ❹ in Fig 5), which is commonly shared by all of the Class I and Class IV DGK homologs. This CSI, in conjunction with the signatures ❷A,B (Fig 4) and the grouping together of the members of these two classes in the phylogenetic tree (Fig 2), provides evidence that the homologs from these two classes are specifically related to each other. In the proximity of Sig ❹, DGK-θ homologs also contain a 1 aa deletion (marked as Sig ❺, shown in light green color). Although, the CSIs ❹ and ❺ are placed in different positions in Fig 5, due to limited sequence similarity observed for the DGK-θ homologs in the vicinity of this indel, it is possible that the indel in DGK-θ (marked as Sig ❺) also occurred in the same position as in the Class I and Class IV DGK isozymes. In that case, the indel marked as ❹ would be a shared characteristic of the Class I, Class IV and Class V DGK isozymes.

**Fig 6:**
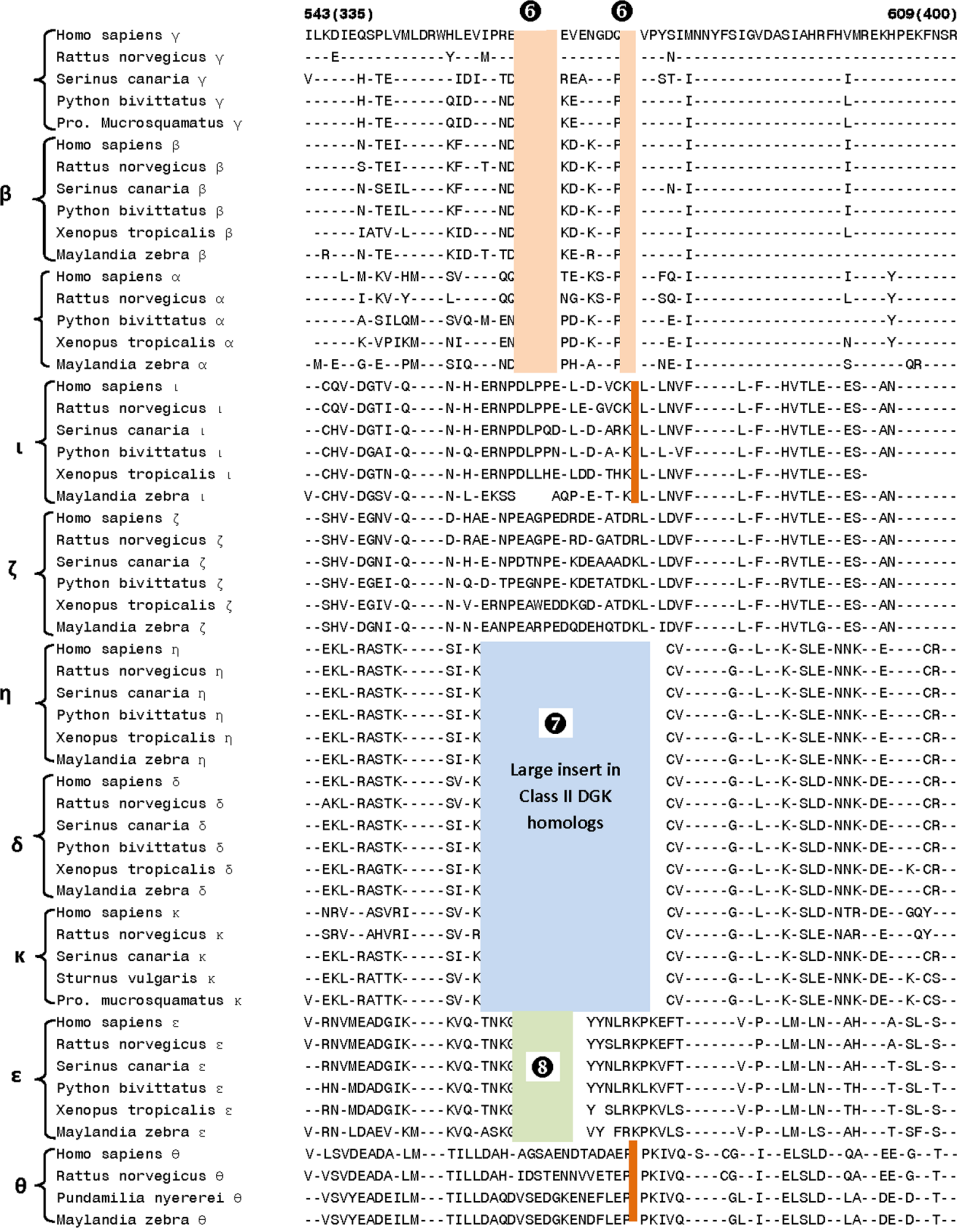
Partial sequence alignment of DGK isozymes showing a region of the catalytic domain where indels of different lengths are present in different DGK isozymes. Signatures marked ❻ are specific for class I DGKs, the large deletion marked ❼ is a shared characteristic of class II DGKs and the CSI marked as ❽ is present in different DGK-θ homologs.

**Fig 7:**
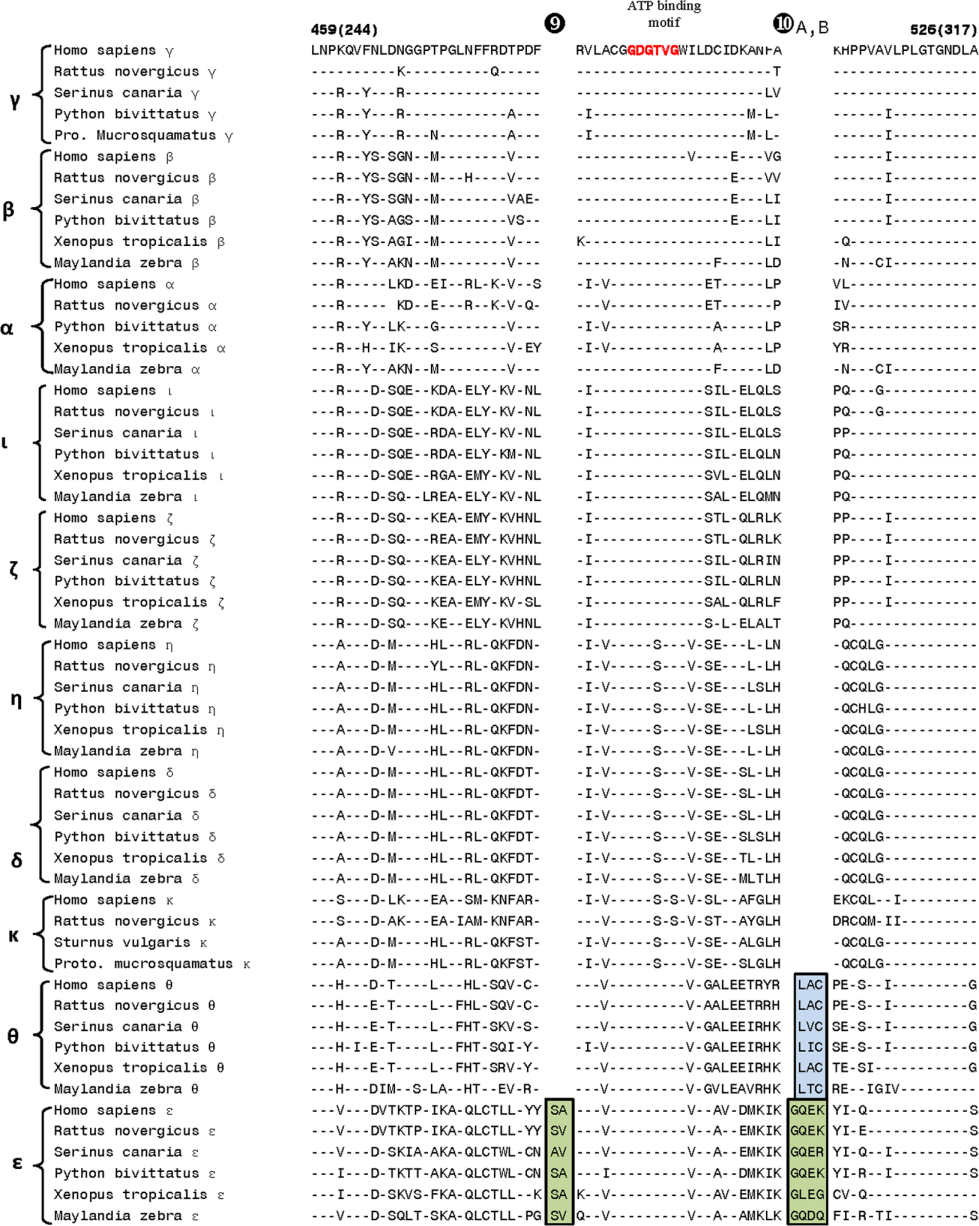
Excerpts from the sequence alignment of DGK isozymes showing a number of CSIs that are specific for the DGK-θ and DGK-ε isozymes. The 2 aa insert marked as Sig❾ is only found in DGK-ε homologs, whereas in a nearby region, both DGK-θ as well as DGK-ε are found to contain CSIs of different lengths in the same position (marked as Sig ❿A, B). These CSIs are located in the proximity of ATP-binding motif (shown in red), which is conserved in all isozymes.

The class II DGK homologs are known to contain a large insert in the middle of the catalytic domain. In the same location, where this large insert is present in the class II homologs, the DGK homologs belonging to other classes are also found to contain smaller CSIs of specific lengths that serve to distinguish them from each other (Fig 6). The Class I DGK homologs contain two different CSIs (a 5 aa deletion and a 2 aa deletion, denoted as Sig ❻) in this region whereas the DGK-ε homologs (class III) all have an 8 aa deletion in this position (marked as Sig ❽). The location of the large insert in the Class II DGK homologs is marked by Sig ❼. In addition, the DGK-θ and DGK-ί isozymes can also be distinguished from others by the presence of 1 aa CSIs (deletions) that appear specific for these isoforms. Lastly, two additional CSIs are present in the catalytic domain in the proximity of the predicted ATP-binding site [2,31,32] shown in the sequence alignment in Fig 7. One of these CSIs, a 2 aa insert, is specific for the DGK-ε homologs (marked as Sig ❾), whereas in a nearby region, both DGK-θ as well as DGK-ε are found to contain CSIs of different lengths in the same position (marked as Sig ❿A, B). However, the sequence of the ATP binding motif (G-X-G-X-X-G) is nearly perfectly conserved in all DGK-isozymes in vertebrates, with the exception of DGK-κ isoform, where a Ser is present in place of the last Gly residue in the mammalian homologs. A number of other residues flanking the ATP-binding motif are also either completely or highly conserved in all DGK isozymes.

### Distribution of DGK isozymes within the domain Eukarya

Much of the work on DGK isozymes has been carried out in mammalian systems and is limited to humans and rodent species. In addition, somework has been carried out on documenting and studying the functions of DGK isozymes in other eukaryotic organisms such as Drosophila, nematodes, slime molds and plants [3,6,10,14–16,33–35]. However, it is unclear whether all of the different DGK isozymes are present in different vertebrate classes and other deeper branching eukaryotic phyla. To gain insights in these regards, we have examined the species distributions of various DGK isozymes in different eukaryotic kingdoms and phyla. These studies were carried out as described in the Methods section and their results are summarized in Table 1. The subphylum vertebrata, which encompasses all of the large animal species, contains five main groups: fishes, amphibians, birds, reptiles and mammals. Of the known DGK isozymes, homologs of DGK- α, -β, -δ, -η, -ζ, -θ and -ε were broadly distributed in all major groups within the vertebrates. While homologs of DGK-κ were mainly found in mammals, birds and some fishes and reptiles, homologs of DGK-γ were not detected in fishes and amphibians. In the invertebrate phyla that were examined (viz. Arthropoda, Nematoda, Mollusca and Cnidarians), homologs that were most similar to DGK-ε, DGK-θ, DGK- ζ, DGK-β and DGK-δ were detected in either all or most species from phyla, whereas homologs for the other DGK isozymes were generally not detected or their presence could not be reliably ascertained. In accordance with earlier studies, homologs showing significant similarity to the DGK isozymes were not detected in Fungi [3,4,6], but in plants, homologs that were most similar to the β- and γ- isozymes were detected [3,10]. Blast searches were also carried out against members of a number of protists phyla (viz. Plasmodium, Euglenezoa, Amoebozoa), which form the deepest branching lineages within the eukaryotes. Except for an isolated hit showing limited similarity to these proteins, the presence of DGK-isozymes in these species could not be reliably ascertained.

**Table 1:** Distribution of different DGK isozymes in the main phyla of vertebrates and other eukaryotes.

Eukaryotic Groups	DAG kinase isoforms									
	α	β	γ	δ	η	κ	ι	ζ	ε	θ
**Mammals (144)**	**+**	**+**	**+**	**+**	**+**	**+**	**+**	**+**	**+**	**+**
**Amphibians (1)**	**+**	**+**	**-**	**+**	**+**	**-**	**+**	**+**	**+**	**+**
**Birds (61)**	**-**^**3**^	**+**	**+**	**+**	**+**	**+**	**+**	**+**	**+**	**+**
**Reptiles (11)**	**+**	**+**	**+**	**+**	**+/-**	**-**^**3**^	**+/-**	**+**	**+**	**+**
**Teleost Fishes (35)**	**+**	**+**	**-**	**+**	**+**	**+/-**	**+**	**+**	**+**	**+**
**Plants (81)**	**-**^**1**^	**+**	**+**	**-**^**1**^	**-**^**1**^	**-**^**1**^	**-**^**1**^	**-**^**1**^	**-**^**1**^	**-**^**1**^
**Fungi (704)**	**-**	**-**^**3**^	**-**^**3**^	**-**	**-**	**-**	**-**	**-**	**-**	**-**
**Plasmodium (45)**	**-**^**1**^	**-**	**+/-**^**2**^	**+/-**^**2**^	**-**^**1**^	**-**^**1**^	**-**^**1**^	**-**^**1**^	**-**^**1**^	**-**^**1**^
**Amoebozoa (16)**	**-**^**1**^	**-**	**+/-**^**1**^	**-**^**1**^	**-**^**1**^	**-**^**1**^	**-**^**1**^	**-**^**1**^	**+/-**^**1**^	**-**^**1**^
**Nematoda (47)**	**-**	**+**	**-**	**+/-**^**2**^	**-**^**1**^	**-**^**1**^	**+/-**^**2**^	**+/-**^**2**^	**+**	**+**
**Mollusca (5)**	**-**	**+**	**-**	**+**	**-**	**-**	**-**	**+**	**+**	**+**
**Cnidarians (5)**	**-**	**-**	**-**^**3**^	**-**^**3**^	**+/-**	**-**	**-**	**+**	**+**	**+**
**Arthropoda (107)**	**-**^**1**^	**+/-**^**2**^	**-**^**1**^	**-**	**+**	**-**	**+/-**^**1**^	**+/-**^**2**^	**+**	**+**
**Platyhelminthes (7)**	**-**	**-**	**-**	**-**	**-**	**-**	**+**	**+/-**	**-**	**+**
**Euglenozoa (47)**	**+/-**^**2**^	**-**^**1**^	**-**^**1**^	**-**^**1**^	**-**^**1**^	**-**^**1**^	**-**^**1**^	**-**^**1**^	**+/-**^**1**^	**-**^**1**^

The presence or absence of different DGK isoforms in the indicated groups was determined based on blastp searches on the annotated genomes in the NCBI nr database as described in the Methods section. The numbers in parenthesis after the group names indicatedthe numbers of annotated genomes that were present in the NCBI nr database from the given group.

(+/-) indicates that only some species may contain the isoform.

^**1**^No isoform was specified however the top 5 hits show limited similarity to the indicated isoform.

^**2**^No isoform was specified however the top 5 hits are most similar to the indicated isoform.

^**3**^Limited similarity (less than 3 hits for the isoform of interest).

## Discussion

Most vertebrate species contain 10 different DGK isozymes which are grouped into 5 different classes or types (Fig 1). The DGKs from different classes are distinguished from each other by the presence or absence of specific functional domains; however, all of them share a large catalytic domain (CD), as well as two cysteine-rich domains. In the simplest of the DGK isozymes, DGK-ε (sole member of class III DGK), the catalytic domain (> 300 aa) makes up nearly 60% of the entire protein and thus it comprises the single largest sequence element specific for the DGK family of proteins. In contrast, the cysteine-rich C1 domains are of short lengths (~ 50 aa), show considerable sequence variation, and they are also present in numerous other proteins including the protein kinase C family of proteins [2–4,12,36]. Due to the prominence and uniqueness of the catalytic domain for the DGK family of proteins, analysis of its sequence provides an important resource for gaining an understanding of the evolution of this protein family as well as functional differences among its members. Our analyses of the catalytic domain sequences presented here have identified many novel sequence features that are specific for different types of DGKs and provide useful insights into the evolution of this protein family.

**Fig 8:**
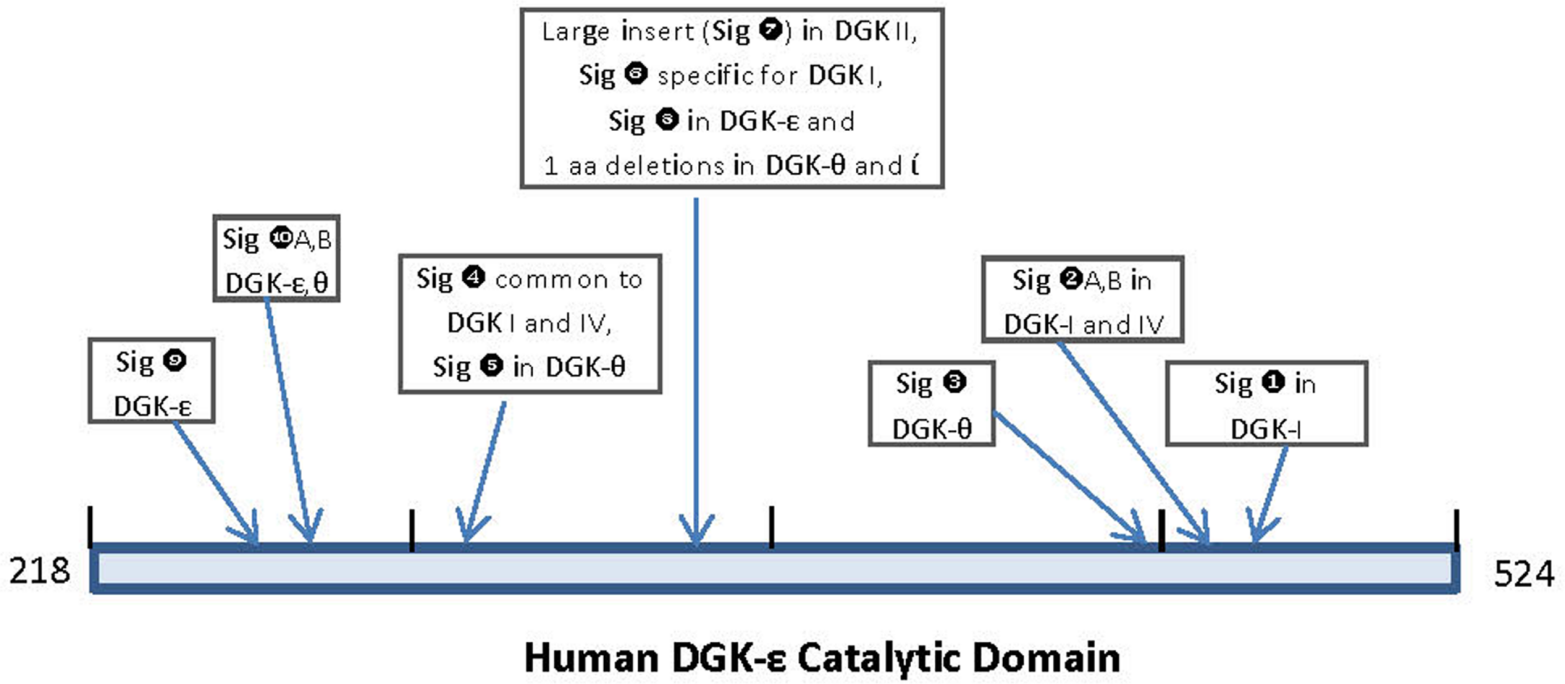
A summary diagram indicating the locations of the CSIs within the catalytic domain that are specific for different DGK classes. The locations of the CSIs are marked using the catalytic domain sequence for the human DGK-ε isoform (accession number NP_003638) as reference.

In a phylogenetic tree based on CD sequences, DGK homologs belonging to different classes formed strongly supported clusters, which were separated by long branches. The observed results indicate strongly that apart from the other known differences in different types of DGK homologs (see [1–6]) their catalytic domain sequences have also diverged considerably from each other and provide clear distinction among different types of DGK homologs. In contrast, the DGK isozymes within each DGK class were tightly clustered in the phylogenetic tree indicating that their catalytic domain sequences are very similar. A major focus of the present work was on identifying novel sequence features within the CD, which are specific for either a particular type of DGK homologs or which are commonly shared by members of different DGK classes. A large number of sequence motifs or signatures, in the forms of CSIs, have been identified in the present work and a summary of the identified CSIs, their DGK class specificity and location within the catalytic domain is provided in Fig 8. Locations of the different CSIs in the CD are indicated using the sequence of the human DGK-ε isoform as reference (accession number NP_003638).

As seen from Fig 8, DGK homologs of all five classes can be clearly distinguished from each other by the presence of specific CSIs that are uniquely found in them. For class I, class III, class IV and class V DGK homologs, multiple CSIs specific for these groups were identified. The described signatures are uniquely present in all members of the indicated classes from vertebrate species (the main focus of the present work) indicating that the genetic changes responsible for these CSIs first occurred in the ancestors of specific DGK isoforms, and they were then retained in the descendent species. Further, according to the definition of the CSI(s) used in our work, the large insert (>300 aa) within the class II DGK members [2–6,12], also represents a CSI (marked as Sig ❼) specific for the class II DGK homologs. The sequence region in the CD where this large CSI is found in class II homologs, it is flanked on both sides by several residues that are conserved in all DGK homologs. Within this conserved region, CSIs of specific lengths (Sig ❻, ❽ and ❽A) which are distinctive characteristics of the other classes of DGK homologs are also found. Based on the differences in the lengths of the CSIs in different classes of DGK homologs, this region is predicted to specify important functional differences in the properties of different classes of DGK homologs. Another noteworthy CSI within the CD is a 16–24 aa insert (Sig ❶ in Fig 3) that is uniquely found in all of the Class I DGK homologs. Some of the identified CSIs were also commonly shared by members from two different DGK types. For example, the CSI in Sig ❹ (Fig 5) is commonly shared by class I and IV, and also possibly class V (Sig ❺), DGK homologs. A closer relationship between class I and IV homologs is also suggested by Sig❷A, B, where inserts that are either 2 aa or 4 aa long are specifically present in these two classes of DGK homologs and by the observed branching pattern of these homologs in the phylogenetic tree. In another instance (Sig❿A, B), CSIs of different lengths (3 aa and 4 aa) in the same position are a uniquely present in theε- and θ- classes of DGK-homologs. Different lengths of CSIs in the same position in specific classes of DGK homologs can results from several possibilities. First, it is possible that independent genetic changes have occurred in the ancestor(s) of specific DGK classes, giving rise to CSIs of different lengths. However, in some cases such as Sig❷A, B (and also Sig❿A, B), it is also possible that initially a genetic change leading to either 2 aa or 4 aa insert occurred in a common ancestor of the class I and IV DGK homologs, and then another insertion or deletion event occurred in the same position in the ancestor of one of these two groups leading to the observed differences in the lengths of the CSIs in these two classes. Although it is difficult to distinguish between these two possibilities, the fact that the class I and class IV DGK homologs also contain another uniquely shared CSI (viz. Sig❹) and they branch together in the phylogenetic tree corroborate the view that the homologs from these two classes are closely related and that the CSI ❷A,B initially occurred in a common ancestor of these two classes.

There is limited understanding at present concerning the overall function of the catalytic domain for eukaryotic DGKs [1–5,31]. Some differences have been recently reported in the activities of different classes of DGKs towards 1-monoacylglycerol and 2-monoacylglycerol [11]. The catalytic domain contains a highly-conserved sequence motif (G-X-G-X-X-G) similar to that present in the ATP-binding sites of other protein kinases [2,31,32]. The importance of this motif for the functioning of DGKs has been established by mutational studies [2,31,37]. The CDs from DGK-ε, -ζ, and -θ proteins exhibit very little DGK activity when expressed as isolated subunits [2]. This and other observations have led to the suggestion that the proper functioning of the CD for mammalian DGKs requires interactions with other motifs (proteins) [1–5,31]. However, our knowledge of the sequence motifs in the CD that may facilitate its interactions with other proteins/effectors molecules, and how different DGK isozymes, or classes of isozymes, differ in this regard is very limited. The lack of structural information for the CD for eukaryotic DGKs is also a major hindrance in understanding its function. In this context, our identification of multiples CSIs within the CD that are specific for different classes of DGK homologs is of much interest and significance. Based on earlier work, the CSIs in protein sequences are known to play important functional roles in the organisms in which they are found [38–42]. The mutational changes in the CSIs affect proper functioning of the proteins as well as cell growth, and both large and small CSIs are indicated to be significant in this regard [38]. Further, extensive work on CSIs show that they are generally located in protein structures in surface loops [39,42–45], which are indicated to play important roles in mediating protein-protein interactions [39,44,46–49]. Based on these observations, it can be hypothesized (predicted) that the CSIs in the CD that are specific for different classes of DGK homologs should also be playing important roles in the interactions of different DGK isozymes with specific proteins (or other target molecules) that may be responsible for the differences in the cellular functions of the different DGK isozymes. Thus, further studies on understanding the cellular functions of the identified CSIs, and the specific interactions that they may be involved in, could provide important insights regarding the biochemical basis of the differences in the functions of different DGK isozymes. It should also be noted that apart from the CSIs that are specific for different classes of DGKs, sequence alignments shown in Figs 2–7 and S1 Fig also contain large numbers of specific amino acid substitutions (or polymorphisms) that are specific for particular classes of DGK homologs. These sequence polymorphisms constitute another type of molecular signature, which are also expected to be of functional significance [12,38]. The DGK family of proteins, due to their key roles in controlling the cellular concentrations of two important signalling molecules and thereby controlling diverse cellular processes, are targets for development of novel therapeutics for treatment of cancer, neuronal, metabolic and inflammatory diseases [9,50–57]. In this context, the CSIs identified in the present work, which are specific for different classes of DGK homologs and predicted to play important roles in the cellular functions of these isoforms, thus provide potentially useful drug targets [58].

The DGK isozymes are primarily found in eukaryotic organisms. Although a protein exhibiting DGK activity is present in many bacteria [4,5,59,60], it shows minimal similarity (15–18%) over a short length, and with multiple intervening gaps, to the eukaryotic DGKs [13] [61]. The bacterial DGK homologs are short in comparison to the eukaryotic proteins and they do not contain any of the canonical sequence features (viz. catalytic domain and cysteine-rich C1 domains), which are shared characteristics of all eukaryotic DGK homologs [2,4,5,12,59]. These observations strongly suggest that the bacterial DGK homologs are evolutionarily unrelated to the eukaryotic counterparts, and that the proteins exhibiting DGK activity have evolved independently in bacteria. In view of the importance of DGK isozymes in cellular signalling processes and their involvement in a wide variety of cellular functions, it is of much interest to know how this large family of proteins has evolved. The phylogenetic approach employed in this work was able to clearly distinguish the five different classes of DGKs; however, as the canonical DGK homologs are only found in eukaryotic organisms, it was difficult to root the tree and determine how members of different DGK classes are related to each other and which of these DGK isozymes is ancestral. The CSIs in protein sequences provide another useful means for understanding the evolutionary relationships among distantly related species; however, in the present instance, due to the absence of a suitable outgroup, it was difficult to establish the ancestral state of the DGK protein sequence and reliably infer whether the observed CSIs are insertions or deletions in the indicated classes of DGK homologs.

Although the present work does not resolve how different classes of DGK proteins have originated from a common ancestor and which of these isoforms is most ancestral, the results presented here show that of the different classes of DGKs, the DGK-ε, -θ and -ζ isoforms are most widely distributed in eukaryotic organisms. In addition to their nearly ubiquitous presence in vertebrate species, the homologs for these isoforms are also indicated to be present in most invertebrate species. In contrast, homologs for the other DGK isozymes were not detected in a number of invertebrate phyla (Table 1). Although more detailed studies are needed to establish with certainty the identity of the DGK isoforms that are present in deep branching eukaryotic phyla, the observed broader distribution of the DGK-ε, DGK-θ and DGK-ζ isoforms within eukaryotic organisms suggests that one of these isoforms likely represent the ancestral form of the protein. Of these isoforms, DGK-ε represents the most rudimentary form of the protein containing all of the main sequence features that are commonly shared by all other DGK family members. Further, DGK-ε is the only isoform which shows specificity for a particular substrate i.e. arachidonoyl-containing diacylglycerol [2,3,6,12,62]. Thus, it is possible that the DGK-ε originated first, and all other DGK isoforms originated from it by acquisition of different domains and other identified signatures. However, the present study is based only on the analyses of the catalytic domain sequences and further work on the analyses of other sequence regions of the DGK protein family could provide additional useful information to clarify the relationships among different DGK isoforms.

## Supporting information

S1 Fig Edited multiple sequence alignment of the catalytic domain for different DGK isozymes that was used for phylogenetic tree construction. (PDF)

S1 Table Sequence information for different DGK homologs used in phylogenetic studies. (PDF)
