# Supplementary material for: Phylogenetic analysis of the diacylglycerol kinase family of proteins and identification of multiple highly-specific conserved inserts and deletions within the catalytic domain that are distinctive characteristics of different classes of DGK homologs
Source: PLoS One. 2017 Aug 22;12(8):e0182758. doi: 10.1371/journal.pone.0182758 (PMC5567653; doi:10.1371/journal.pone.0182758)
Supplement: S1 Fig — (PDF) [file pone.0182758.s001.pdf]

S1 Fig. Edited multiple sequence alignment of the catalytic domain for different DGK isozymes that was used for phylogenetic tree construction.

```
Xenopustropicalis-alpha             PLLVFVNPKSGGKQGERVLRKFQYLLNPRQVHNLIKGGPSPGLNFFRDVP
Maylandiazebra-alpha                PLLVFVNPKSGGKQGERVLRKFQFLLNPRQVYNLSNGGPGPGLSFFRNLK
Homosapiens-gamma                   PLLVLVNPKSGGRQGERILRKFHYLLNPKQVFNLDNGGPTPGLNFFRDTP
Rattusnorvegicus-gamma              PLLVLVNPKSGGRQGERILQKFHYLLNPKQVFNLDKGGPTPGLNFFQDTP
Serinuscanaria-gamma                PLLVFVNPKSGGRQGERVLRKFHYLLNPRQVYNLDRGGPTPGLNFFRDTP
Sturnusvulgaris-gamma               PLLVFVNPKSGGRQGERVLRKFHYLLNPRQVYNLDRGGPTPGLNFFRDTP
Pythonbivittatus-gamma              PLLVFVNPKSGGRQGERVHRKFHYLLNPRQVYNLDRGGPTPGLNFFRDAP
Protobothropsmucrosquamatus-ga      PLLVFVNPKSGGRQGERVHRKFHYILNPRQVYNLDRGGPNPGLNFFRDAP
Rattusnorvegicus-zeta               PLLVFVNPKSGGNQGAKIIQSFLWYLNPRQVFDLSQGGPREALEMYRKVH
Serinuscanaria-zeta                 PLLVFVNPKSGGNQGAKIIQSFMWYLNPRQVFDLSQGGPKEALELYRKVH
Sturnusvulgaris-zeta                PLLVFVNPKSGGNQGAKIIQSFMWYLNPRQVFDLSQGGPKEALELYRKVH
Protobothropsmucrosquamatus-ze      PLLVFVNPKSGGNQGTKIFQSFMWYLNPRQVFDLSQGGPKEALELYRKVH
Pythonbivittatus-zeta               PLLVFVNPKSGGNQGTKIFQSFMWYLNPRQVFDLSQGGPKEALELYRKVH
Xenopustropicalis-zeta              PVLVFVNPKSGGNQGVKILQSFMWYLNPRQVFDLSQGGPKEALEMYRKVP
Maylandiazebra-zeta                 PLLVFVNPKSGGNQGTKILQSFMWYLNPRQVFDLSQGGPKEGLELYRKVH
Pundamilianyererei-zeta             PLLVFVNPKSGGNQGTKILQSFMWYLNPRQVFDLSQGGPKEGLELYRKVH
Homosapiens-epsilon-other           PLIILANSRSGTNMGEGLLGEFRILLNPVQVFDVTKTPPIKALQLCTLLP
Rattusnorvegicus-epsilon-other      PLIILANSRSGTNMGEGLLGEFKMLLNPVQVFDVTKTPPIKALQLCTLLP
Pythonbivittatus-epsilon            PVIILVNTRSGNNMGETLMGQLKILLNPIQVFDLTKTTPAKALQLCTWLP
Protobothropsmucrosquamatus-ep      PVIILVNTRSGNNMGETLIGQFKILLNPIQVFDLTKTAPAKALQLCTWLP
Serinuscanaria-epsilon              PVMVLANTRSGNNMGEIFLGEFKMLLNPVQVFDLSKIAPAKALQLCTWLP
Sturnusvulgaris-epsilon             PVMVLANTRSGNNMGETLLGEFKMLLNPVQVFDLSKIGPAKALQLCTWLP
Xenopustropicalis-epsilon           PLIVLANTRSGNNMGEALLSEFKGLLNPIQVFDLSKVSPFKALQLCTLLP
Pundamilianyererei-epsilon          PVLVLANTRSGNNMGEVLLGEFRTLLNPVQVFDLSQLTPSKALQLCTLLP
Homosapiens-alpha                   PLLVFVNPKSGGKQGQRVLWKFQYILNPRQVFNLLKDGPEIGLRLFKDVP
Rattusnorvegicus-alpha              PLLVFINPKSGGKQGQSVLWKFQYILNPRQVFN-LKDGPEPGLRFFKDVP
Pythonbivittatus-alpha              PLLVFVNPKSGGKQGERVLRKFQYLLNPRQVYNLLKGGPGPGLNFFRDVP
Protobothropsmucrosquamatus-al      PLLVFVNPKSGGKQGERVLRKFQYLLNPRQVYNLLKGGPGPGLNFFRDIP
Maylandiazebra-epsilon              PVLVLANTRSGNNMGEVLLGEFRTLLNPVQVFDLSQLTPSKALQLCTLLP
Sturnusvulgaris-theta               PLLVFVNPKSGGLKGRDLLYSFRKLLNPHQVFELTNGGPLPGFHTFSKVP
Serinuscanaria-theta                PLLVFVNPKSGGLKGRDLLYSFRKLLNPHQVFELTNGGPLPGFHTFSKVP
Rattusnorvegicus-theta              PLLVFVNPKSGGLKGRELLCSFRKLLNPHQVFELTNGGPLPGFHLFSQVP
Homosapiens-theta                   PLLVFVNPKSGGLKGRDLLCSFRKLLNPHQVFDLTNGGPLPGLHLFSQVP
Protobothropsmucrosquamatus-th      PLLVFVNAKSGSLKGRDLLHSFRKLLNPHQIFELTNGGPLPGFHAFSQIP
Xenopustropicalis-theta             PLLVFVNPKSGGLKGRDLLYSFRKLLNPHQVFELTNGGPLPGFHTFSRVP
Pundamilianyererei-theta            PLLVFVNPKSGGLKGRELLYGFRKLLNPHQVFDIMNGSPLAGLHTFREVP
Maylandiazebra-theta                PLLVFVNPKSGGLKGRELLYGFRKLLNPHQVFDIMNGSPLAGLHTFREVP
Rattusnorvegicus-beta               PLLVFVNPKSGGKQGERIYRKFQYLLNPRQVYSLSGNGPMPGLHFFRDVP
Xenopustropicalis-beta              PLLVFVNPKSGGKQGERIHRKFQYLLNPRQVYSLAGIGPMPGLNFFRDVP
Protobothropsmucrosquamatus-be      PLLVFVNPKSGGKQGERIYRKFQYLLNPRQVYSLAGNGPMPGLNFFRDVS
Serinuscanaria-beta                 PLLVFVNPKSGGKQGERIYRKFQYLLNPRQVYSLSGNGPMPGLNFFRDVA
Pundamilianyererei-beta             PLLVFVNPKSGGKQGERIYRKFQYLLNPRQVYNLAKNGPMPGLNFFRDVP
Maylandiazebra-beta                 PLLVFVNPKSGGKQGERIYRKFQYLLNPRQVYNLAKNGPMPGLNFFRDVP
Homosapiens-beta                    PLLVFVNPKSGGKQGERIYRKFQYLLNPRQVYSLSGNGPMPGLNFFRDVP
Pythonbivittatus-delta              PLLVFVNSKSGDNQGVKFLRRFKQLLNPAQVFDLMNGGPHLGLRLFQKFD
Protobothropsmucrosquamatus-de      PLLVFVNSKSGDNQGVKFLRRFKQLLNPAQVFDLMNGGPHLGLRLFQKFD
Sturnusvulgaris-delta               PLLVFVNSKSGDNQGVKFLRRFKQLLNPAQVFDLMNGGPHLGLRLFQKFD
Xenopustropicalis-delta             PLLVFVNSKSGDNQGVKFLRRFKQLLNPAQVFDLMNGGPHLGLRLFQKFD
Homosapiens-eta-other               PLLVFVNSKSGDNQGVKFLRRFKQLLNPAQVFDLMNGGPHLGLRLFQKFD
Serinuscanaria-delta                PLLVFVNSKSGDNQGVKFLRRFKQLLNPAQVFDLMNGGPHLGLRLFQKFD
Pundamilianyererei-delta            PLLVFVNSKSGDNQGVKFLRRFKQLLNPAQVFDLMNGGPHLGLRLFQKFD
Maylandiazebra-delta                PLLVFVNSKSGDNQGVKFLRRFKQLLNPAQVFDLMNGGPHLGLRLFQKFD
Homosapiens-delta-other             PLLVFVNSKSGDNQGVKFLRRFKQLLNPAQVFDLMNGGPHLGLRLFQKFD
Rattusnorvegicus-delta              PLLVFVNSKSGDNQGVKFLRRFKQLLNPAQVFDLMNGGPHLGLRLFQKFD
Rattusnorvegicus-eta                PLLVFVNSKSGDNQGVKFLRRFKQLLNPAQVFDLMNGGPYLGLRLFQKFD
Maylandiazebra-eta                  PLLVFVNSKSGDNQGVKFLRRFKQLLNPAQVFDLVNGGPHLGLRLFQKFD
Serinuscanaria-eta                  PLLVFVNSKSGDNQGVKFLRRFKQSLNPAQVFDLMNGGPHLGLRLFQKFD
Sturnusvulgaris-eta                 PLLVFVNSKSGDNQGVKFLRRFKQSLNPAQVFDLMNGGPHLGLRLFQKFD
Xenopustropicalis-eta               PLLVFVNSKSGDNQGVKFLRRFKQLLNPAQVFDLMNGGPHLGLRLFQKFD
Pythonbivittatus-eta                PLLVFVNSKSGDNQGVKFLRRFKQSLNPAQVFDLMNGGPHLGLRLFQKFD
Protobothropsmucrosquamatus-et      PLLVFVNSKSGDNQGVKFLRRFKQSLNPAQVFDLMNGGPHLGLRLFQKFD
```

```
Serinuscanaria-kappa                PLLAFVNSKSGDNQGVKFLRKFKQFLNPAQVFDLMNGGPHLGLRLFQKFS
Sturnusvulgaris-kappa               PLLAFVNSKSGDNQGVKFLRKFKQFLNPAQVFDLMNGGPHLGLRLFQKFS
Protobothropsmucrosquamatus-ka      PLLAFVNSKSGDNQGVKFLRKFKQFLNPAQVFDLMNGGPHLGLRLFQKFS
Homosapiens-kappa                   PLLIFINSKSGDHQGIVFLRKFKQYLNPSQVFDLLKGGPEAGLSMFKNFA
Bostaurus]Kappa                     PLLIFINSKSGDHQGVVFLRKFKQYLNPSQVFDLSKGGPEAGLCMFKNFA
Rattusnorvegicus]kappa              PLLIFINSKSGDHQGIIFLRKFKQYLNPSQVFDLAKGGPEAGIAMFKNFA
Maylandiazebra-iota                 PILVFVNPKSGGNQGAKVLQMFMWILNPRQVFDLSQGGLREALELYRKVP
Pundamilianyererei-iota             PILVFVNPKSGGNQGAKVLQMFMWILNPRQVFDLSQGGLREALELYRKVP
Homosapiens-iota                    PLLVFVNPKSGGNQGTKVLQMFMWYLNPRQVFDLSQEGPKDALELYRKVP
Rattusnorvegicus-iota               PLLVFVNPKSGGNQGTKVLQMFMWYLNPRQVFDLSQEGPKDALELYRKVP
Sturnusvulgaris-iota                PLLVFVNPKSGGNQGTKVLQMFMWYLNPRQVFDLSQEGPRDALELYRKVP
Serinuscanaria-iota                 PLLVFVNPKSGGNQGTKVLQMFMWYLNPRQVFDLSQEGPRDALELYRKVP
Protobothropsmucrosquamatus-io      PLLVFVNPKSGGNQGTKVLQMFMWYLNPRQVFDLSQEGPRDALELYRKMP
Homosapiens-zeta                    PLLVFVNPKSGGNQGAKIIQSFLWYLNPRQVFDLSQGGPKEALEMYRKVH
                                    *:: : *.:**   *  .   :   *** *:..        .:

Xenopustropicalis-alpha             --EYRILVCGGDGTVGWILDAIDKANLPYR----PPVAVLPLGTGNDLAR
Maylandiazebra-alpha                --EYRILVCGGDGTVGWILDAIDKGNLLVR----PPVAVLPLGTGNDLAR
Homosapiens-gamma                   --DFRVLACGGDGTVGWILDCIDKANFAKH----PPVAVLPLGTGNDLAR
Rattusnorvegicus-gamma              --DFRVLACGGDGTVGWILDCIDKANFTKH----PPVAVLPLGTGNDLAR
Serinuscanaria-gamma                --DFRVLACGGDGTVGWILDCIDKANLVKH----PPVAVLPLGTGNDLAR
Sturnusvulgaris-gamma               --DFRVLACGGDGTVGWILDCIDKANLVKH----PPVAVLPLGTGNDLAR
Pythonbivittatus-gamma              --DFRILACGGDGTVGWILDCIDKMNLAKH----PPVAILPLGTGNDLAR
Protobothropsmucrosquamatus-ga      --DFRILACGGDGTVGWILDCIDKMNLAKH----PPVAILPLGTGNDLAR
Rattusnorvegicus-zeta               --NLRILACGGDGTVGWILSTLDQLRLKPP----PPVAILPLGTGNDLAR
Serinuscanaria-zeta                 --NLRILACGGDGTVGWILSILDQLRINPP----PPVAILPLGTGNDLAR
Sturnusvulgaris-zeta                --NLRILACGGDGTVGWILSILDQLRINPP----PPVAILPLGTGNDLAR
Protobothropsmucrosquamatus-ze      --NLRILACGGDGTVGWILSILDQLRLNPP----PPVAILPLGTGNDLAR
Pythonbivittatus-zeta               --NLRILACGGDGTVGWILSILDQLRLNPP----PPVAILPLGTGNDLAR
Xenopustropicalis-zeta              --SLRILACGGDGTVGWILSALDQLRLFPP----PPVAILPLGTGNDLAR
Maylandiazebra-zeta                 --NLRILACGGDGTVGWILSCLDELALTPQ----PPVAVLPLGTGNDLAR
Pundamilianyererei-zeta             --NLRILACGGDGTVGWILSCLDELALTPQ----PPVAVLPLGTGNDLAR
Homosapiens-epsilon-other           YYSARVLVCGGDGTVGWVLDAVDDMKIKGQEKYIPQVAVLPLGTGNDLSN
Rattusnorvegicus-epsilon-other      YYSVRVLVCGGDGTVGWVLDAIDEMKIKGQEKYIPEVAVLPLGTGNDLSN
Pythonbivittatus-epsilon            CNSARVLICGGDGTVGWVLDAIDDMKIKGQEKYIPRVAILPLGTGNDLSN
Protobothropsmucrosquamatus-ep      SNSARVLVCGGDGTVGWVLDAIDSMKIKGQERSVPRVAILPLGTGNDLSN
Serinuscanaria-epsilon              CNAVRVLVCGGDGTVGWVLDAIDEMKIKGQERYIPQVAILPLGTGNDLSN
Sturnusvulgaris-epsilon             CNTVRVLVCGGDGTVGWVLDAIDEMKIKGQERYIPQVAILPLGTGNDLSN
Xenopustropicalis-epsilon           DKSAKVLVCGGDGTVGWVLDAVDEMKIKGLEGCVPQVAVLPLGTGNDLAN
Pundamilianyererei-epsilon          PGSVQVLVCGGDGTVGWVLDAIDAMKLKGQDQFIPRVTILPLGTGNDLSN
Homosapiens-alpha                   --DSRILVCGGDGTVGWILETIDKANLPVL----PPVAVLPLGTGNDLAR
Rattusnorvegicus-alpha              --QFRVLVCGGDGTVGWILETIDKANFPIV----PPVAVLPLGTGNDLAR
Pythonbivittatus-alpha              --DFRILVCGGDGTVGWILDAIDKANLPSR----PPVAVLPLGTGNDLAR
Protobothropsmucrosquamatus-al      --NFRILVCGGDGTVGWILDAIDKANLPSR----PPVAVLPLGTGNDLAR
Maylandiazebra-epsilon              PGSVQVLVCGGDGTVGWVLDAIDAMKLKGQDQFIPRVTILPLGTGNDLSN
Sturnusvulgaris-theta               --SFRVLVCGGDGTVGWVLGALEEIRHK-LVCSEPSVAILPLGTGNDLGR
Serinuscanaria-theta                --SFRVLVCGGDGTVGWVLGALEEIRHK-LVCSEPSVAILPLGTGNDLGR
Rattusnorvegicus-theta              --CFRVLVCGGDGTVGWVLAALEETRRH-LACPEPSVAILPLGTGNDLGR
Homosapiens-theta                   --CFRVLVCGGDGTVGWVLGALEETRYR-LACPEPSVAILPLGTGNDLGR
Protobothropsmucrosquamatus-th      --YFRILVCGGDGTVGWVLGALEEIRHK-LSWPEPSVAILPLGTGNDLGR
Xenopustropicalis-theta             --YFRVLVCGGDGTVGWVLGALEEIRHK-LACTEPSIAVLPLGTGNDLGR
Pundamilianyererei-theta            --RFRVLVCGGDGTVGWVLGVLEAVRHK-LTCREPPIGIVPLGTGNDLAR
Maylandiazebra-theta                --RFRVLVCGGDGTVGWVLGVLEAVRHK-LTCREPPIGIVPLGTGNDLAR
Rattusnorvegicus-beta               --DFRVLACGGDGTVGWILDCIEKANVVKH----PPVAILPLGTGNDLAR
Xenopustropicalis-beta              --DFKVLACGGDGTVGWILDCIDKANLIKQ----PPVAVLPLGTGNDLAR
Protobothropsmucrosquamatus-be      --DFRVLACGGDGTVGWILDCIEKANLIKH----PPVAILPLGTGNDLAR
Serinuscanaria-beta                 --EFRVLACGGDGTVGWILDCIEKANLIKH----PPVAILPLGTGNDLAR
Pundamilianyererei-beta             --DFRVLACGGDGTVGWILDFIDKANLDKN----PPVCILPLGTGNDLAR
Maylandiazebra-beta                 --DFRVLACGGDGTVGWILDFIDKANLDKN----PPVCILPLGTGNDLAR
Homosapiens-beta                    --DFRVLACGGDGTVGWVLDCIEKANVGKH----PPVAILPLGTGNDLAR
Pythonbivittatus-delta              --TFRILVCGGDGSVGWVLSEIDSLSLHKQ----CQLGVLPLGTGNDLAR
Protobothropsmucrosquamatus-de      --TFRILVCGGDGSVGWVLSEIDSLSLHKQ----CQLGVLPLGTGNDLAR
Sturnusvulgaris-delta               --TFRILVCGGDGSVGWVLSEIDSLNLHKQ----CQLGVLPLGTGNDLAR
Xenopustropicalis-delta             --TFRILVCGGDGSVGWVLSEIDTLNLHKQ----CQLGVLPLGTGNDLAR
Homosapiens-eta-other               --NFRILVCGGDGSVGWVLSEIDKLNLNKQ----CQLGVLPLGTGNDLAR
```

```
Serinuscanaria-delta                --TFRILVCGGDGSVGWVLSEIDSLNLHKQ----CQLGVLPLGTGNDLAR
Pundamilianyererei-delta            --TFRILVCGGDGSVGWVLSEIDMLTLHKQ----CQLGVLPLGTGNDLAR
Maylandiazebra-delta                --TFRILVCGGDGSVGWVLSEIDMLTLHKQ----CQLGVLPLGTGNDLAR
Homosapiens-delta-other             --TFRILVCGGDGSVGWVLSEIDSLNLHKQ----CQLGVLPLGTGNDLAR
Rattusnorvegicus-delta              --TFRILVCGGDGSVGWVLSEIDSLNLHKQ----CQLGVLPLGTGNDLAR
Rattusnorvegicus-eta                --NFRILVCGGDGSVGWVLSEIDKLNLHKQ----CQLGVLPLGTGNDLAR
Maylandiazebra-eta                  --NFRILVCGGDGSVGWVLSEIDKLNLHKQ----CQLGVLPLGTGNDLAR
Serinuscanaria-eta                  --NFRILVCGGDGSVGWVLSEIDKLSLHKQ----CQLGVLPLGTGNDLAR
Sturnusvulgaris-eta                 --NFRILVCGGDGSVGWVLSEIDKLSLHKQ----CQLGVLPLGTGNDLAR
Xenopustropicalis-eta               --NFRILVCGGDGSVGWVLSEIDKLSLHKQ----CQLGVLPLGTGNDLAR
Pythonbivittatus-eta                --NFRILVCGGDGSVGWVLSEIDKLNLHKQ----CHLGVLPLGTGNDLAR
Protobothropsmucrosquamatus-et      --NFRILVCGGDGSVGWVLSEIDKLNLHKQ----CHLGVLPLGTGNDLAR
Serinuscanaria-kappa                --TFRILVCGGDGSVGWVLSEIDALGLHKQ----CQLGVLPLGTGNDLAR
Sturnusvulgaris-kappa               --TFRILVCGGDGSVGWVLSEIDALGLHKQ----CQLGVLPLGTGNDLAR
Protobothropsmucrosquamatus-ka      --TFRILVCGGDGSVGWVLSEIDSLGLHKQ----CQLGVLPLGTGNDLAR
Homosapiens-kappa                   --RFRILVCGGDGSVSWVLSLIDAFGLHEK----CQLAVIPLGTGNDLAR
Bostaurus]Kappa                     --RFRVVVCGGDGSVNWVLSLIDAFGLHEQ----CQLAVIPLGTGNDLAR
Rattusnorvegicus]kappa              --RFRVLVCGGDGSVSWVLSTIDAYGLHDR----CQMAIIPLGTGNDLAR
Maylandiazebra-iota                 --NLRILACGGDGTVGWILSALDELQMNPQ----PPVAVLPLGTGNDLAR
Pundamilianyererei-iota             --NLRILACGGDGTVGWILSALDELQMNPQ----PPVAVLPLGTGNDLAR
Homosapiens-iota                    --NLRILACGGDGTVGWILSILDELQLSPQ----PPVGVLPLGTGNDLAR
Rattusnorvegicus-iota               --NLRILACGGDGTVGWILSILDELQLSPQ----PPVGVLPLGTGNDLAR
Sturnusvulgaris-iota                --NLRILACGGDGTVGWILSILDELQLSPP----PPVAVLPLGTGNDLAR
Serinuscanaria-iota                 --NLRILACGGDGTVGWILSILDELQLSPP----PPVAVLPLGTGNDLAR
Protobothropsmucrosquamatus-io      --NLRILACGGDGTVGWILSILDELQLNPQ----PPVAVLPLGTGNDLAR
Homosapiens-zeta                    --NLRILACGGDGTVGWILSTLDQLRLKPP----PPVAILPLGTGNDLAR
                                        ::: *****:*.*:*  ::             : ::********..

Xenopustropicalis-alpha             CLSWGGGYDGENLMK-FLKDIEKSVPIKMDRWNIEVIYFSIGVDASIAHR
Maylandiazebra-alpha                CLRWGGGYDGEDLNR-ILKDIEGSSQVLMDRWSVQVIYFSIGVDASIAHR
Homosapiens-gamma                   CLRWGGGYEGGSLTK-ILKDIEQSPLVMLDRWHLEVIYFSIGVDASIAHR
Rattusnorvegicus-gamma              CLRWGGGYEGGSLTK-ILKEIEQSPLVMLDRWYLEVMYFSIGVDASIAHR
Serinuscanaria-gamma                CLRWGGGYEGGNLMK-VLKDIEHSTEVMLDRWHIDIIYFSIGVDASIAHR
Sturnusvulgaris-gamma               CLRWGGGYEGGNLMK-VLKDIEHSTEVMLDRWHIDIIYFSIGVDASIAHR
Pythonbivittatus-gamma              CLRWGGGYEGGNLIK-ILKDIEHSTEVMLDRWQIDVIYFSIGVDASIAHR
Protobothropsmucrosquamatus-ga      CLRWGGGYEGGNLIK-ILKDIEHSTEVMLDRWQIDVIYFSIGVDASIAHR
Rattusnorvegicus-zeta               TLNWGGGYTDEPVSK-ILSHVEEGNVVQLDRWDLRAEYFSLGFDAHVTLE
Serinuscanaria-zeta                 TLNWGGGYTDEPLSK-ILSHVEDGNIVQLDRWNLHVEYFSLGFDARVTLE
Sturnusvulgaris-zeta                TLNWGGGYTDEPVSK-ILSHVEDGNIVQLDRWNLHVEYFSLGFDARVTLE
Protobothropsmucrosquamatus-ze      TLNWGGGYTDEPLSK-ILSHVEEGEIVQLDRWNLQVEYFSLGFDARVTLE
Pythonbivittatus-zeta               TLNWGGGYTDEPLSK-ILSHVEEGEIVQLDRWNLLVEYFSLGFDARVTLE
Xenopustropicalis-zeta              TLNWGGGYTDEPLSK-ILSHVEEGIVVQLDRWNLVVEYFSLGFDAHVTLE
Maylandiazebra-zeta                 TLNWGGGYTDEPLSK-ILSHVEEGTVVQLDRWNLLVEYFSLGFDAHVTLE
Pundamilianyererei-zeta             TLNWGGGYTDEPLSK-ILSHVEEGTVVQLDRWNLLVEWDSLPFXXXVTLE
Homosapiens-epsilon-other           TLGWGTGYAGEIPVAQVLRNVMEADGIKLDRWKVQVTYFSVGPDALMALN
Rattusnorvegicus-epsilon-other      TLGWGTGYAGEIPVAQVLRNVMEADGIKLDRWKVQVTYFSIGPDALMALN
Pythonbivittatus-epsilon            TLGWGAGYAGEVPVEQILHNIMDADGIKLDRWKVQVTYFSVGPDALMALN
Protobothropsmucrosquamatus-ep      TLGWGAGYAGDVPVEQILHNIMDADEIQLDRWKVQVIYFSVGPDALMALN
Serinuscanaria-epsilon              TLGWGAGYAGEVPVEQILRNVMEADGIKLDRWKVQVTYFSVGPDALMALN
Sturnusvulgaris-epsilon             TLGWGAGYAGEAPVEQILRNVMEADGIKLDRWKVQVTYFSVGPDALMALN
Xenopustropicalis-epsilon           TLGWGAGYAGDVPVEQILRNIMDADGIKLDRWKVQVTYFSVGPDALMALN
Pundamilianyererei-epsilon          TLGWGAGYAGEIPVEQVLRNILDAEVVKMDRWKVQVAYFSVGPDALMALS
Homosapiens-alpha                   CLRWGGGYEGQNLAK-ILKDLEMSKVVHMDRWSVEVIYFSIGVDASIAHR
Rattusnorvegicus-alpha              CLRWGRGYEGENLRK-ILKDIEISKVVYLDRWLLEVIYFSIGVDASIAHR
Pythonbivittatus-alpha              CLRWGGGYDGENLVK-ILKDIEASSILQMDRWSVQVMYFSIGVDASIAHR
Protobothropsmucrosquamatus-al      CLRWGGGYDGENLVK-ILKDIEASSIIQMDRWSVQVMYFSIGVDASIAHR
Maylandiazebra-epsilon              TLGWGAGYAGEIPVEQVLRNILDAEVVKMDRWKVQVAYFSVGPDALMALS
Sturnusvulgaris-theta               VLRWGAGYSGEDPYS-ILVSVDEADDVLMDRWTILLDYCGLGIDAELSLD
Serinuscanaria-theta                VLRWGAGYSGEDPYS-ILVSVDEADDVLMDRWTILLDYCGLGIDAELSLD
Rattusnorvegicus-theta              VLRWGAGYSGEDPFS-VLVSVDEADAVLMDRWTILLDYCGIGIDAELSLD
Homosapiens-theta                   VLRWGAGYSGEDPFS-VLLSVDEADAVLMDRWTILLDYCGIGIDAELSLD
Protobothropsmucrosquamatus-th      VLRWGAGYSGEDPYS-ILISVDEADDVLMDRWTILLDYCGLGIDAELSLG
Xenopustropicalis-theta             VLRWGAGYSGEDPYS-ILISVNEAENVLMDRWTILLDYCGIGIDAELSLD
Pundamilianyererei-theta            ILRWGPGYSGEDPYH-ILVSVYEADEILMDRWTILLDYFGLGIDAELSLD
Maylandiazebra-theta                ILRWGPGYSGEDPYH-ILVSVYEADEILMDRWTILLDYFGLGIDAELSLD
```

```
Rattusnorvegicus-beta               CLRWGGGYEGENLMK-ILKDIESSTEIMLDRWKFEVTYFSIGVDASIAHR
Xenopustropicalis-beta              CLRWGGGYEGENLMK-FLKDIEIATVVLLDRWKIDVIYFSIGVDASIAHR
Protobothropsmucrosquamatus-be      CLRWGGGYEGENLLK-ILKDIENSTEILLDRWKFEVIYFSIGVDASIAHR
Serinuscanaria-beta                 CLRWGGGYEGENLMK-ILKDIENSSEILLDRWKFEVIYFSIGVDASIAHR
Pundamilianyererei-beta             CLRWGGGYEGESLLK-ILRDIENSTEVMLDRWKIDVTYFSIGVDASIAHR
Maylandiazebra-beta                 CLRWGGGYEGESLLK-ILRDIENSTEVMLDRWKIDVTYFSIGVDASIAHR
Homosapiens-beta                    CLRWGGGYEGENLMK-ILKDIENSTEIMLDRWKFEVIYFSIGVDASIAHR
Pythonbivittatus-delta              VLGWGSACDDDTQLPQILEKLERASTKMLDRWSIMVYYFGIGLDAKISLD
Protobothropsmucrosquamatus-de      VLGWGSACDDDTQLPQILEKLERASTKMLDRWSIMVYYFGIGLDAKISLD
Sturnusvulgaris-delta               VLGWGSACDDDTQLPQILEKLERASTKMLDRWSIMVYYFGIGLDAKISLD
Xenopustropicalis-delta             VLGWGSACDDDTQLPQILEKLERAGTKMLDRWSIMVYYFGIGLDAKISLD
Homosapiens-eta-other               VLGWGGSYDDDTQLPQILEKLERASTKMLDRWSIMTYYFGIGLDAKISLE
Serinuscanaria-delta                VLGWGSACDDDTQLPQILEKLERASTKMLDRWSIMVYYFGIGLDAKISLD
Pundamilianyererei-delta            VLGWGSACDDDTQLPQILEKLERASTKMLDRWSIMVYYFGIGLDAKISLD
Maylandiazebra-delta                VLGWGSACDDDTQLPQILEKLERASTKMLDRWSIMVYYFGIGLDAKISLD
Homosapiens-delta-other             VLGWGSACDDDTQLPQILEKLERASTKMLDRWSVMAYYFGIGLDAKISLD
Rattusnorvegicus-delta              VLGWGSACDDDTQLPQILAKLERASTKMLDRWSVMAYYFGIGLDAKISLD
Rattusnorvegicus-eta                VLGWGGSYDDDTQLPQILEKLERASTKMLDRWSIMTYYFGIGLDAKISLE
Maylandiazebra-eta                  VLGWGPSCDDDTQLPQILEKLERASTKMLDRWSIMTYYFGIGLDAKISLE
Serinuscanaria-eta                  VLGWGGSCDDDTQLPQILEKLERASTKMLDRWSIMSYYFGIGLDAKISLE
Sturnusvulgaris-eta                 VLGWGGSCDDDTQLPQILEKLERASTKMLDRWSIMSYYFGIGLDAKISLE
Xenopustropicalis-eta               VLGWGASCDDDTQLPQILEKLERASTKMLDRWSIMSYYFGIGLDAKISLE
Pythonbivittatus-eta                VLGWGGSCDDDTQLPQILEKLERASTKMLDRWSIMSYYFGIGLDAKISLE
Protobothropsmucrosquamatus-et      VLGWGGSCDDDTQLPQILEKLERASTKMLDRWSIMSYYFGIGLDAKISLE
Serinuscanaria-kappa                VLGWGSLCDDDTQLLQILEKLERATTKMLDRWSVLTYYFGIGLDAKISLE
Sturnusvulgaris-kappa               VLGWGSLCDDDTQLLQILEKLERATTKMLDRWSVLTYYFGIGLDAKISLE
Protobothropsmucrosquamatus-ka      VLGWGSLCDDDTQLLQVLEKLERATTKMLDRWSVLTYYFGIGLDAKISLE
Homosapiens-kappa                   VLGWGAFWNKSKSPLDILNRVEQASVRILDRWSVMIRYFGIGLDAKISLD
Bostaurus]Kappa                     VLGWGAFWNKNKSPLNILNRVEQAGVRTLDRWSVMIRYFGIGLDAKISLE
Rattusnorvegicus]kappa              VLGWGAFWSKGKSPLEILSRVEQAHVRILDRWSVMIRYFGIGLDAKISLE
Maylandiazebra-iota                 TLNWGGGYTDEPVSK-VLCHVEDGSVVQLDRWNLLVEYFSLGFDAHVTLE
Pundamilianyererei-iota             TLNWGGGYTDEPVSK-VLCHVEDGSVVQLDRWNLLVEYFSLGFDAHVTLE
Homosapiens-iota                    TLNWGGGYTDEPVSK-ILCQVEDGTVVQLDRWNLHVEYFSLGFDAHVTLE
Rattusnorvegicus-iota               TLNWGGGYTDEPVSK-ILCQVEDGTIVQLDRWNLHVEYFSLGFDAHVTLE
Sturnusvulgaris-iota                TLNWGGGYTDEPVSK-ILCHVEDGTIVQLDRWNLQVEYFSLGFDAHVTLE
Serinuscanaria-iota                 TLNWGGGYTDEPVSK-ILCHVEDGTIVQLDRWNLHVEYFSLGFDAHVTLE
Protobothropsmucrosquamatus-io      TLNWGGGYTDEPVAK-ILCHVEDGAIVQLDRWNLQVEYFSLGFDAHVTLE
Homosapiens-zeta                    TLNWGGGYTDEPVSK-ILSHVEEGNVVQLDRWDLHAEYFSLGFDAHVTLE
                                     * **           .*  :  .    :*** .   : .:     ::

Xenopustropicalis-alpha             FHNMREKYPEKFNSRMKNKLWYFEFATSETVFSTCKKLKEVITIECCGSP
Maylandiazebra-alpha                FHTMREKHPQKFNSRMKNKLWYFEFATSETISASCKKLSENLTIECCGTP
Homosapiens-gamma                   FHVMREKHPEKFNSRMKNKLWYFEFGTSETFAATCKKLHDHIELECDGVG
Rattusnorvegicus-gamma              FHVMREKHPEKFNSRMKNKLWYFEFGTSETFAATCKKLHDHIELECDGVE
Serinuscanaria-gamma                FHIMREKHPEKFNSRMKNKLWYFEFGTSETFAATCKKLHDYVEVECDGTL
Sturnusvulgaris-gamma               FHIMREKHPEKFNSRMKNKLWYFEFGTSETFAATCKKLHDYVEVECDGTV
Pythonbivittatus-gamma              FHLMREKHPEKFNSRMKNKLWYFEFGTTETFAATCKKLHDYVEIECDGTV
Protobothropsmucrosquamatus-ga      FHLMREKHPEKFNSRMKNKLWYFEFGTTETFSATCKKLHDYVEIECDGTV
Rattusnorvegicus-zeta               FHESREANPEKFNSRFRNKMFYAGTAFSDFLMGSSKDLAKHIRVVCDGMD
Serinuscanaria-zeta                 FHESREANPEKFNSRFRNKMFYAGTAFSDFLTGSSKDLAKHVRLVCDGID
Sturnusvulgaris-zeta                FHESREANPEKFNSRFRNKMFYAGTAFSDFLTGSSKDLAKHVRLVCDGTD
Protobothropsmucrosquamatus-ze      FHESREANPEKFNSRFRNKMFYAGTAFSDFLMGSSKDLAKHIKVVCDGTD
Pythonbivittatus-zeta               FHESREANPEKFNSRFRNKMFYAGTAFSDFLMGSSRDLAKHIKVVCDGTD
Xenopustropicalis-zeta              FHESREANPEKFNSRFRNKMFYAGTAVSDFFTGSSRDLAKHIRVVCDGVD
Maylandiazebra-zeta                 FHESREANPEKFNSRFRNKMFYAGTAFSDFLMGSSKDLSKHIKVVCDGTD
Pundamilianyererei-zeta             FHESREANPEKFNSRFRNKMFYAGTAFSDFLMGSSKDLSKHIKVVCDGTD
Homosapiens-epsilon-other           FHAHREKAPSLFSSRILNKAVYLFYGTKDCLVQECKDLNKKVELELDGER
Rattusnorvegicus-epsilon-other      FHAHREKAPSLFSSRILN------------------------KLELDGER
Pythonbivittatus-epsilon            FHTHREKTPSLFTSRIINKAVYFFYGTKDCLVQECKDLNKKIELELDGEK
Protobothropsmucrosquamatus-ep      FHVHREKTPSLFTSRIINKAVYFFYGTKDCLVQECKDLNKKIELELDGEK
Serinuscanaria-epsilon              FHAHREKTPSLFSSRIINKAVYFFYGTKDCLVQECKDLNKKVELELDGER
Sturnusvulgaris-epsilon             FHAHREKTPSLFSSRIINKAVYFFYGTKDCLVQQCKDLNKKVELELDGER
Xenopustropicalis-epsilon           FHTHREKTPSLFSSRLVNKAVYLFYGTKDCLVQECKDLNKKVELELDGER
Pundamilianyererei-epsilon          FHAHREKTPSFFSSRIINKAVYFLYGTRDCLVQECKDLDKRIELELDGER
Homosapiens-alpha                   FHIMREKYPEKFNSRMKNKLWYFEFATSESIFSTCKKLEESLTVEICGKP
```

```
Rattusnorvegicus-alpha            FHLMREKYPEKFNSRMKNKLWYLEFATSESIFSTCKKLEESVTVEICGKL
Pythonbivittatus-alpha            FHVMREKYPEKFNSRMKNKLWYFEFATSETIFATCKKLKECLSIECCGQS
Protobothropsmucrosquamatus-al    FHVMREKYPEKFNSRMKNKLWYFEFATSETLFATCKKLKECLSLECCGQS
Maylandiazebra-epsilon            FHAHREKTPSFFSSRIINKAVYFLYGTRDCLVQECKDLDKRIELELDGER
Sturnusvulgaris-theta             FHHAREEEPGKFNSRFHNKGVYVKVGLQK--ISHTRNLHKDIKLQVDQHE
Serinuscanaria-theta              FHHAREEEPGKFNSRFHNKGVYVKVGLQK--ISHTRNLHKDIKLQVDQHE
Rattusnorvegicus-theta            FHQAREEEPGKFTSRFHNKGVYVRVGLQK--ISHSRSLHKEIRLQVEQQE
Homosapiens-theta                 FHQAREEEPGKFTSRLHNKGVYVRVGLQK--ISHSRSLHKQIRLQVERQE
Protobothropsmucrosquamatus-th    FHHAREEEPDKFNSRLHNKGVYVKVGLQK--MSHTRSLHKEIKLQMDQHE
Xenopustropicalis-theta           FHHAREIEPGKFNSRFHNKGVYVKAGLQK--LSHNRNLHRDMKLQVDQQE
Pundamilianyererei-theta          FHLAREDEPDKFTSRFHNKGVYVKVGLQK--ISHTRSLHKELQLHVDGQE
Maylandiazebra-theta              FHLAREDEPDKFTSRFHNKGVYVKVGLQK--ISHTRSLHKELQLHVDGQE
Rattusnorvegicus-beta             FHIMREKHPEKFNSRMKNKFWYFEFGTSETFSATCKKLHESVEIECDGVQ
Xenopustropicalis-beta            FHLMREKHPEKFNSRMKNKFWYFEFGTSETFSATCKKLHEAIEIECDGIQ
Protobothropsmucrosquamatus-be    FHIMREKHPEKFNSRMKNKFWYFEFGTSETFSATCKKLHESLEIECDGIQ
Serinuscanaria-beta               FHIMREKHPEKFNSRMKNKFWYFEFGTSETFSATCKKLHESVEIECDGIQ
Pundamilianyererei-beta           FHVMREKHPEKFNSRTKNKLWYFEFGTSETFSATCKKLHDFLEVECDGVT
Maylandiazebra-beta               FHVMREKHPEKFNSRTKNKLWYFEFGTSETFSATCKKLHDFLEVECDGVT
Homosapiens-beta                  FHIMREKHPEKFNSRMKNKFWYFEFGTSETFSATCKKLHESVEIECDGVQ
Pythonbivittatus-delta            FNNKRDEHPEKCRSRTKNMMWYGVLGTKELLHRTYKNLEQKVLLECDGRP
Protobothropsmucrosquamatus-de    FNNKRDEHPEKCRSRTKNMMWYGVLGTKELLHRTYKNLEQKVLLECDGRP
Sturnusvulgaris-delta             FNNKRDEHPEKCRSRTKNMMWYGVLGTKELLHRTYKNLEQKVLLECDGRP
Xenopustropicalis-delta           FNNKRDEHPKKCRSRTKNMMWYGVLGTKELLQRTYKNLEQRVLLECDGVS
Homosapiens-eta-other             FNNKREEHPEKCRSRTKNLMWYGVLGTRELLQRSYKNLEQRVQLECDGQY
Serinuscanaria-delta              FNNKRDEHPEKCRSRTKNMMWYGVLGTKELLHRTYKNLEQKVLLECDGRP
Pundamilianyererei-delta          FNNKRDEHPEKCRSRTKNMMWYGVLGTKELLHRTYKNLEQRVLLECDGRP
Maylandiazebra-delta              FNNKRDEHPEKCRSRTKNMMWYGVLGTKELLHRTYKNLEQRVLLECDGRP
Homosapiens-delta-other           FNNKRDEHPEKCRSRTKNMMWYGVLGTKELLHRTYKNLEQKVLLECDGDP
Rattusnorvegicus-delta            FNNKRDEHPEKCRSRTKNMMWYGVLGTKELLHRTYRNLEQKVLLECDGRP
Rattusnorvegicus-eta              FNNKREEHPEKCRSRTKNLMWYGVLGTRELLQRSYKNLEQRVQLECDGQY
Maylandiazebra-eta                FNNKREEHPEKCRSRTKNMMWYGVLGTKELLQRTYKNLEQKVQLECDGQY
Serinuscanaria-eta                FNNKREEHPEKCRSRTKNMMWYGVLGTKELLQRTYKNLEQKVQLECDGQY
Sturnusvulgaris-eta               FNNKREEHPEKCRSRTKNMMWYGVLGTKELLQRTYKNLEQKVQLECDGQY
Xenopustropicalis-eta             FNNKREEHPEKCRSRTKNMMWYGVLGTKELLQRTYKNLEQKVQLECDGQY
Pythonbivittatus-eta              FNNKREEHPEKCRSRTKNMMWYGVLGTKELLQRTYKNLEQKVQLECDGQY
Protobothropsmucrosquamatus-et    FNNKREVHPEKCRSRTKNMMWYGVLGTKELLQRTYKNLEQKVQLECDGQY
Serinuscanaria-kappa              FNNKRDEHPKKCSSRTKNMMWYGVLGTKELLQRTYKNLEQRVQLECDGVP
Sturnusvulgaris-kappa             FNNKRDEHPKKCSSRTKNMMWYGVLGTKELLQRTYKNLEQRVQLECDGVP
Protobothropsmucrosquamatus-ka    FNNKRDEHPKKCSSRTKNMMWYGVLGTKELLQRTYKNLEQRVQLECDGVP
Homosapiens-kappa                 FNTRRDEHPGQYNSRLKNKMWYGLLGTKELLQRSYRKLEERVHLECDGET
Bostaurus]Kappa                   FNTRRDEHPGQYNSRFKNKMWYGLLGSKELFQRSYRKLEERVHLECDGEA
Rattusnorvegicus]kappa            FNARREEHPEQYNSRLKNKIWYGLLGSKELLQRSYRKLEERIHLECDGEA
Maylandiazebra-iota               FHESREANPEKFNSRFRNKMFYAGAAFSDFLQRSSRDLSKHVRVVCDGTD
Pundamilianyererei-iota           FHESREANPEKFNSRFRNKMFYAG---------------------CDGTD
Homosapiens-iota                  FHESREANPEKFNSRFRNKMFYAGAAFSDFLQRSSRDLSKHVKVVCDGTD
Rattusnorvegicus-iota             FHESREANPEKFNSRFRNKMFYAGAAFSDFLQRSSRDLSKHVKVVCDGTD
Sturnusvulgaris-iota              FHESREANPEKFNSRFRNKMFYAGAAFTDFLQRSSRDLSKHVKVVCDGTD
Serinuscanaria-iota               FHESREANPEKFNSRFRNKMFYAGAAFTDFLQRSSRDLSKHVKVVCDGTD
Protobothropsmucrosquamatus-io    FHESREANPEKFNSRFRNKMFYAGAAFSDFLQRSSRDLSKHVKVVCDGTD
Homosapiens-zeta                  FHESREANPEKFNSRFRNKMFYAGTAFSDFLMGSSKDLAKHIRVVCDGMD
                                  *:  *:  *    ** *

Xenopustropicalis-alpha           LLEGIAVINIPSMHGGSNLWGETKKHWGEPRNASCHN---APEAITDPDA
Maylandiazebra-alpha              LLEGVAILNIPSMHGGSNLWGETKK--VDTKGLTAQEE---PEVIVNPEI
Homosapiens-gamma                 VLEGIAILNIPSMYGGTNLWGENKKNRAVIRESRKG--------VTDPKE
Rattusnorvegicus-gamma            VLEGIAILNIPSMYGGTNLWGETKKNRAVIRESRKS--------VTDPKE
Serinuscanaria-gamma              LLEGIAVLNIPSMYGGSNLWGETKKQRGYNRLGKKVPEKVPGTVVTDAKE
Sturnusvulgaris-gamma             LLEGIAVLNIPSMYGGSNLWGETKKQRGYNRLGKKAPEKVPGTVVTDAKE
Pythonbivittatus-gamma            LLEGIAVLNIPSMYGGSNLWGETKKQRSYNRMSKKIPEKLQSSVVTDAKE
Protobothropsmucrosquamatus-ga    LLEGIAVLNIPSMYGGSNLWGETKKQRSYNRMSKKIPEKLQYSVVTDAKE
Rattusnorvegicus-zeta             LPQCIVFLNIPRYCAGTMPWGHPGE------------------------H
Serinuscanaria-zeta               LPQCLVFLNIPRYCAGTMPWGNPGE------------------------H
Sturnusvulgaris-zeta              LPQCLVFLNIPRYCAGTMPWGNPGE------------------------H
Protobothropsmucrosquamatus-ze    LPQCLVFLNIPRYCAGTMPWGNPGE------------------------H
Pythonbivittatus-zeta             LPQCLVFLNIPRYCAGTMPWGNPGE------------------------H
```

```
Xenopustropicalis-zeta              LLQCLVFLNIPRYCAGTMPWGNPGE------------------------H
Maylandiazebra-zeta                 LLQCLVFLNIPRYCAGTTPWGNPSE------------------------H
Pundamilianyererei-zeta             LLQCLVFLNIPRYCAGTTPWGNPSE------------------------H
Homosapiens-epsilon-other           VLEGIIVLNIGYWGGGCRLWEGMGD-------------------------
Rattusnorvegicus-epsilon-other      VLEGVIVLNIGYWGGGCRLWEGVGD-------------------------
Pythonbivittatus-epsilon            ILEGIIVLNIAYWGGGCRLWEGMGD-------------------------
Protobothropsmucrosquamatus-ep      ILEGIIVLNIAYWGGGCRLWEGMGD-------------------------
Serinuscanaria-epsilon              ILEGIIVLNIGYWGGGCRLWEGMGD-------------------------
Sturnusvulgaris-epsilon             ILEGIIVLNIGYWGGGCRLWEGMGD-------------------------
Xenopustropicalis-epsilon           ILEGIVVLNIGYWGGGCRLWEGMGD-------------------------
Pundamilianyererei-epsilon          VLEGIIVCNIGYWGGGCRLWEGMGD-------------------------
Homosapiens-alpha                   LLEGIAVLNIPSMHGGSNLWGDTRRPHGDIYGINQALG-ATAKVITDPDI
Rattusnorvegicus-alpha              LLEGIAVLNIPSMHGGSNLWGDTKRPHGDTCGINQALG-SVAKIITDPDI
Pythonbivittatus-alpha              LLSGIAVLNIPSMHGGSNLWGETKRPLGEAAARSTAGGAAQPQVITEAEI
Protobothropsmucrosquamatus-al      LLSGVAVLNIPSMHGGSNLWGETKRPLGEAAARSTAGGAAQPQVITEAEI
Maylandiazebra-epsilon              VLEGIIVCNIGYWGGGCRLWEGMGD-------------------------
Sturnusvulgaris-theta               VIEGLIFINIPSWGSGADLWGSESD-------------------------
Serinuscanaria-theta                VIEGLIFINIPSWGSGADLWGSESD-------------------------
Rattusnorvegicus-theta              VIEGLIFINIPSWGSGADLWGSDSD-------------------------
Homosapiens-theta                   VIEGLIFINIPSWGSGADLWGSDSD-------------------------
Protobothropsmucrosquamatus-th      VIEGLIFINIPSWGSGADLWGSDND-------------------------
Xenopustropicalis-theta             VIEGLIFLNIPSWGSGADLWGSDND-------------------------
Pundamilianyererei-theta            VIEGLIFLNIPSWGSGADLWGSEVD-------------------------
Maylandiazebra-theta                VIEGLIFLNIPSWGSGADLWGSEVD-------------------------
Rattusnorvegicus-beta               ILQGIAILNIPSMHGGSNLWGESKKKRSHRRIEKKGSDKR--PTLTDAKE
Xenopustropicalis-beta              MLEGIAILNIPSMHGGSNLWGETKKRRSNRRTDKKNSDKR--TTVTDAKE
Protobothropsmucrosquamatus-be      ILEGIAILNIPSMHGGSNLWGETKKRRSHRRFEKNRPDKR--ITVTDAKE
Serinuscanaria-beta                 LLEGIAILNIPSMHGGSNLWGETKKRRSHRRTEKKRSDKR--TTVTDAKE
Pundamilianyererei-beta             LLEGIAILNIPSMHGGSNLWGESKKRRGYRKGGKKGQDKR--TPVLDPKE
Maylandiazebra-beta                 LLEGIAILNIPSMHGGSNLWGESKKRRGYRKGGKKGQDKR--TPVLDPKE
Homosapiens-beta                    ILEGIAILNIPSMHGGSNLWGESKKRRSHRRIEKKGSDKR--TTVTDAKE
Pythonbivittatus-delta              ILQGIAVLNIPSYAGGTNFWGGTKE------------------------D
Protobothropsmucrosquamatus-de      ILQGIAVLNIPSYAGGTNFWGGTKE------------------------D
Sturnusvulgaris-delta               ILQGIAVLNIPSYAGGTNFWGGTKE------------------------D
Xenopustropicalis-delta             ILQGIAVLNIPSYAGGTNFWGGSKE------------------------N
Homosapiens-eta-other               ILQGIAVLNIPSYAGGTNFWGGTKE------------------------D
Serinuscanaria-delta                ILQGIAVLNIPSYAGGTNFWGGTKE------------------------D
Pundamilianyererei-delta            ILQGIAVLNIPSYAGGTNFWGGTKE------------------------D
Maylandiazebra-delta                ILQGIAVLNIPSYAGGTNFWGGTKE------------------------D
Homosapiens-delta-other             SLQGIAVLNIPSYAGGTNFWGGTKE------------------------D
Rattusnorvegicus-delta              ILQGIAVLNIPSYAGGTNFWGGTKE------------------------D
Rattusnorvegicus-eta                ILQGIAVLNIPSYAGGTNFWGGTKE------------------------D
Maylandiazebra-eta                  ILQGIAVLNIPSYAGGTNFWGGTKE------------------------D
Serinuscanaria-eta                  ILQGIAVLNIPSYAGGTNFWGGTKE------------------------D
Sturnusvulgaris-eta                 ILQGIAVLNIPSYAGGTNFWGGTKE------------------------D
Xenopustropicalis-eta               ILQGIAVLNIPSYAGGTNFWGGTKE------------------------D
Pythonbivittatus-eta                ILQGIAVLNIPSYAGGTNFWGGTKE------------------------D
Protobothropsmucrosquamatus-et      ILQGIAVLNIPSYAGGTNFWGGTKE------------------------D
Serinuscanaria-kappa                ILQGIAVLNIPSYAGGINFWGGTKE------------------------D
Sturnusvulgaris-kappa               ILQGIAVLNIPSYAGGINFWGGTKE------------------------D
Protobothropsmucrosquamatus-ka      ILQGIAVLNIPSYAGGINFWGGTKE------------------------D
Homosapiens-kappa                   ILQGIVVLNITSYAGGINFWGSNTA------------------------T
Bostaurus]Kappa                     ILQGIVVLNITSYAGGVNFWGSSTA------------------------T
Rattusnorvegicus]kappa              VLQGIVVLNITSYAGGVNFWGSNTA------------------------T
Maylandiazebra-iota                 LFQCIVFLNIPRYCAGTMPWGNTGD------------------------H
Pundamilianyererei-iota             LFQCIVFLNIPRYCAGTMPWGNTGD------------------------H
Homosapiens-iota                    LFQCIVFLNIPRYCAGTMPWGNPGD------------------------H
Rattusnorvegicus-iota               LFQCIVFLNIPRYCAGTMPWGNPGD------------------------H
Sturnusvulgaris-iota                LFQCIVFLNIPRYCAGTMPWGNPGD------------------------H
Serinuscanaria-iota                 LFQCIVFLNIPRYCAGTMPWGNPGD------------------------H
Protobothropsmucrosquamatus-io      LFQCIVFLNIPRYCAGTMPWGNPGD------------------------H
Homosapiens-zeta                    LPQCVVFLNIPRYCAGTMPWGHPGE------------------------H
                                      . : . **    .*   *

Xenopustropicalis-alpha             LKSCVQDLSDKRLEVVGLEGAIEMGQIYTGLKSAGKRLATCSELTIRTTK
```

```
Maylandiazebra-alpha                LKVTSQDLSDRRLEVVGLEGAMEMGQIYTGLKSA-VRLAKTSQITIRTKK
Homosapiens-gamma                   LKFCVQDLSDQLLEVVGLEGAMEMGQIYTGLKSAGRRLAQCASVTIRTNK
Rattusnorvegicus-gamma              LKCCVQDLSDQLLEVVGLEGAMEMGQIYTGLKSAGRRLAQCSSVTIRTKK
Serinuscanaria-gamma                LKFCVQDLSDHLLEVVGLEGAMEMGQIYTGLKSAGKRLAQCSSVIIRTSK
Sturnusvulgaris-gamma               LKFCVQDLSDHLLEVVGLEGAMEMGQIYTGLKSAGKRLAQCSSVIIRTSK
Pythonbivittatus-gamma              LKFCVQDLSDQLLEVVGLEGAMEMGQIYTGLKSAGKRLAQCSSVAIRTTK
Protobothropsmucrosquamatus-ga      LKFCVQDLSDQLLEVVGLEGAMEMGQIYTGLKSAGKRLAQCSSVAIRTTK
Rattusnorvegicus-zeta               HDFEPQRHDDGYLEVIGFTMTSLAALQVGG---HGERLTQCREVLLTTAK
Serinuscanaria-zeta                 HDFEPQRHDDGCIEVIGFTMTSLAALQVGG---HGERLCQCRQVVLTTSK
Sturnusvulgaris-zeta                HDFEPQRHDDGCIEVIGFTMTSLAALQVGG---HGERLCQCRQVVLTTSK
Protobothropsmucrosquamatus-ze      HDFEPQRHDDGCLEVIGFTMTSLAALQVGG---HGERLHQCREVVLTTSK
Pythonbivittatus-zeta               HDFEPQRHDDGCLEVIGFTMTSLAALQVGG---HGERLHQCREVVLTTSK
Xenopustropicalis-zeta              HDFEPQRHDDGCIEVIGFTMASLAALQVGG---HGERLHQCREVLLTTSK
Maylandiazebra-zeta                 HDFEPQRHDDGYIEVIGFTMTSLATLQVGG---HGERLNQCREVILTTTK
Pundamilianyererei-zeta             HDFEPQRHDDGYIEVIGFTMTSLATLQVGG---HGERLNQCREVILTTTK
Homosapiens-epsilon-other           ETYPLARHDDGLLEVVGVYGSFHCAQIQVKLANP-FRIGQAHTVRLILKC
Rattusnorvegicus-epsilon-other      ETYPLARHDDGLLEVVGVYGSFHCAQIQVKLANP-FRIGQAHTVRLTLKC
Pythonbivittatus-epsilon            ELYPLARHDDGLLEVVGVNGSFHCAQIQVKLANP-VRLGQAHTVRLILKN
Protobothropsmucrosquamatus-ep      ELYPRARHDDGLLEVVGVNGSFHCAQIQVKLANP-VRLGQAHTVRLILKN
Serinuscanaria-epsilon              EPYPLARHDDGLLEVVGVHGSFHCAQIQVKLANP-VRLGQAHTVRLILKS
Sturnusvulgaris-epsilon             EPYPLARHDDGLLEVVGVHGSFHCAQIQVKLANP-VRLGQAHTVRLILKS
Xenopustropicalis-epsilon           EPYPLSRHDDGLLEVVGVYGSFHCAQIQVKLANP-VRLGQAHTVRLLLKS
Pundamilianyererei-epsilon          EPCPPTRLDDGLLEVVGVFGSFHCAQIQVKLANP-VRLGQAHTVRLVLKS
Homosapiens-alpha                   LKTCVPDLSDKRLEVVGLEGAIEMGQIYTKLKNAGRRLAKCSEITFHTTK
Rattusnorvegicus-alpha              LKTCVPDMSDKRLEVVGIEGVIEMGQIYTRLKSAGHRLAKCSEITFQTTK
Pythonbivittatus-alpha              LKNCVQDLSDRRMEVVGLEGVFEMGQIYTGLKNAGTRLAKCSEITLRTFK
Protobothropsmucrosquamatus-al      LKNCVQDLTDRRMEVVGLEGVFEMGQIYTGLKNAGKRLAKCSEITLRTFK
Maylandiazebra-epsilon              EPCPPTRLDDGLLEVVGVFGSFHCAQIQVKLANP-VRLGQAHTVRLVLKS
Sturnusvulgaris-theta               NRFEKPRIDDGLLEVVGVTGVVHMGQVQGGFRSG-IRIAQGSYFRVTLLK
Serinuscanaria-theta                NRFEKPRIDDGLLEVVGVTGVVHMGQVQGGFRSG-IRIAQGSYFRVTLLK
Rattusnorvegicus-theta              SRFEKPRIDDGLLEVVGVTGVVHMGQVQGGLRSG-IRIAQGSYFRVTLLK
Homosapiens-theta                   TRFEKPRMDDGLLEVVGVTGVVHMGQVQGGLRSG-IRIAQGSYFRVTLLK
Protobothropsmucrosquamatus-th      SRFEKPKIDDGLLEVVGVTGVVHMGQVQGGLRSG-IRIAQGSYFRVTLRK
Xenopustropicalis-theta             NRYGKPRIDDGLLEVVGVTGVVHMGQVQGGLRSG-IRIAQGSYFRVTLLK
Pundamilianyererei-theta            SRYEKPSIDDGLLEVVGVTGVVHMGQVQSGFRSG-IRIAQGNYIRLTLRK
Maylandiazebra-theta                SRYEKPSIDDGLLEVVGVTGVVHMGQVQSGFRSG-IRIAQGNYIRLTLRK
Rattusnorvegicus-beta               LKFASQDLSDQLLEVVGLEGAMEMGQIYTGLKSAGRRLAQCSSVVIRTSK
Xenopustropicalis-beta              LKFAAQDLSDQLLEVVGLEGAMEMGQIYTGLKSAGRRLAQCSSVVLRTSK
Protobothropsmucrosquamatus-be      LKFACQDLSDQLVEVVGLEGAMEMGQIYTGLKSAGRRLSQCSSVFIRTSK
Serinuscanaria-beta                 LKFVCQDLSDQLMEVVGLEGAMEMGQIYTGLKSAGRRLAQCSSVVIRTSK
Pundamilianyererei-beta             LMFAVQDLSDQLLEVVGLEGAMEMGQIYTGLKSAGRRLAQCSSVTIRTSK
Maylandiazebra-beta                 LMFAVQDLSDQLLEVVGLEGAMEMGQIYTGLKSAGRRLAQCSSVTIRTSK
Homosapiens-beta                    LKFASQGLSDQLLEVVGLEGAMEMGQIYTGLKSAGRRLAQCSCVVIRTSK
Pythonbivittatus-delta              DTFTAPSFDDKILEVVAVFGSMQMA-VSRVINLQHHRIAQCRTVKIEILG
Protobothropsmucrosquamatus-de      DTFTAPSFDDKILEVVAVFGSMQMA-VSRVINLQHHRIAQCRTVKIAILG
Sturnusvulgaris-delta               DTFTAPSFDDKILEVVAVFGSMQMA-VSRVINLQHHRIAQCRTVKIAILG
Xenopustropicalis-delta             DTFAAPSFDDKILEVVAVFGSMQMA-VSRVIKLQHHRIAQCRTVKITVLG
Homosapiens-eta-other               DIFAAPSFDDKILEVVAIFDSMQMA-VSRVIKLQHHRIAQCRTVKITIFG
Serinuscanaria-delta                DTFTAPSFDDKILEVVAVFGSMQMA-VSRVINLQHHRIAQCRTVKIAILG
Pundamilianyererei-delta            DTFTAPSFDDKILEVVAVFGSMQMA-VSRVINLQHHRIAQCRTVKITILG
Maylandiazebra-delta                DTFTAPSFDDKILEVVAVFGSMQMA-VSRVINLQHHRIAQCRTVKITILG
Homosapiens-delta-other             DTFAAPSFDDKILEVVAVFGSMQMA-VSRVIRLQHHRIAQCRTVKISILG
Rattusnorvegicus-delta              DTFAAPSFDDKILEVVAVFGSMQMA-VSRVIKLQHHRIAQCRTVKISILG
Rattusnorvegicus-eta                DIFAAPSFDDKILEVVAVFDSVQMA-VSRVIKLQHHRIAQCRTVKITIFG
Maylandiazebra-eta                  DIFCAPSFDDKILEVVAVFGSMQMA-VSRVIKLQHHRIAQCRTVKITILG
Serinuscanaria-eta                  DIFGAPSFDDKILEVVAVFGSMQMA-VSRVIKLQHHRIAQCRSVKITILG
Sturnusvulgaris-eta                 DIFGAPSFDDKILEVVAVFGSMQMA-VSRVIKLQHHRIAQCRSVKITILG
Xenopustropicalis-eta               DIFGAPSFDDKILEVVAVFGSMQMA-VSRVIKLQHHRIAQCRSVKITILG
Pythonbivittatus-eta                DIFGAPSFDDKILEVVAVFGSMQMA-VSRVIKLQHHRIAQCRTVKITILG
Protobothropsmucrosquamatus-et      DIFGAPSFDDKILEVVAVFGSMQMA-VSRVIKLQHHRIAQCRAVKITILG
Serinuscanaria-kappa                NNFGAPSFDDKKLEVVAVFGSIQMA-VSRVINLQHHRIAQCRVVKITIRG
Sturnusvulgaris-kappa               NNFGAPSFDDKKLEVVAVFGSIQMA-VSRVINLQHHRIAQCRVVKITIRG
Protobothropsmucrosquamatus-ka      SNFGAPSFDDKKLEVVAVFGSIQMA-VSRVINLQHHRIAQCRMVKITIRG
Homosapiens-kappa                   TEYEAPAIDDGKLEVVAIFGSVQMA-MSRIINLHHHRIAQCHEVMITIDG
Bostaurus]Kappa                     TEYEAPAIDDGKLEVVAIFGSVQMA-MSRIINLYHHRIAQCREVMITING
```

```
Rattusnorvegicus]kappa              TEYEAPAIDDGKLEVVAIFGSVQMA-MSRIINLHHHRIAQCREVIITIDG
Maylandiazebra-iota                 RDFEPQRHDDGCIEVIGFTMASLAALQVGG---HGERLHQCREVILTTYK
Pundamilianyererei-iota             RDFEPQRHDDGCIEVIGFTMASLAALQVGG---HGERLHQCREVILTTYK
Homosapiens-iota                    HDFEPQRHDDGYIEVIGFTMASLAALQVGG---HGERLHQCREVMLLTYK
Rattusnorvegicus-iota               HDFEPQRHDDGYIEVIGFTMASLAALQVGG---HGERLHQCREVMLLTYK
Sturnusvulgaris-iota                RDFEPQRHDDGYIEVIGFTMASLAALQVGG---HGERLHQCREVTLLTYK
Serinuscanaria-iota                 RDFEPQRHDDGYIEVIGFTMASLAALQVGG---HGERLHQCREVTLLTYK
Protobothropsmucrosquamatus-io      REFEPQRHDDGFIEVIGFTMASLAALQVGG---HGERLHQCREVTLLTYK
Homosapiens-zeta                    HDFEPQRHDDGYLEVIGFTMTSLAALQVGG---HGERLTQCREVVLTTSK
                                             *  :**:..                  *:     . .

Xenopustropicalis-alpha             P---LPMQIDGEPWMQPPCTIKITHKNQAPML
Maylandiazebra-alpha                A---LPMQIDGEPWMQPPCTIQITHKNQACML
Homosapiens-gamma                   L---LPMQVDGEPWMQPCCTIKITHKNQAPMM
Rattusnorvegicus-gamma              L---LPMQVDGEPWMQPPCMIKITHKNQAPMM
Serinuscanaria-gamma                L---LPMQVDGEPWMQPSCTVKITHKSQVPML
Sturnusvulgaris-gamma               L---LPMQVDGEPWMQPSCTVKITHKSQVPML
Pythonbivittatus-gamma              L---LPMQVDGEPWMQPPCTIKITHKSQVPML
Protobothropsmucrosquamatus-ga      L---LPMQVDGEPWMQPPCTIKITHKSQVPML
Rattusnorvegicus-zeta               A---IPVQVDGEPCKLAASRIRIALRNQATMV
Serinuscanaria-zeta                 A---IPMQVDGEPCKLAASCIHISLRNQANMV
Sturnusvulgaris-zeta                A---IPMQVDGEPCKLAASCIHISLRNQANMV
Protobothropsmucrosquamatus-ze      A---IPMQVDGEPCKLGASCIRISLRNQANMV
Pythonbivittatus-zeta               A---IPMQVDGEPCKLGASCIRISLRNQANMV
Xenopustropicalis-zeta              S---IPMQVDGEPCKLGPSVIKISLRNQANLV
Maylandiazebra-zeta                 P---LPVQVDGEPCRLAPSVIHISLRNQANMV
Pundamilianyererei-zeta             P---LPVQVDGEPCRLAPSVIHISLRNQANMV
Homosapiens-epsilon-other           S--MMPMQVDGEPWAQGPCTVTITHKTHAMML
Rattusnorvegicus-epsilon-other      SRMPMPMQVDGEPWAQGPCTVTITHKTHALML
Pythonbivittatus-epsilon            S--KMPMQVDGEPWAQGPCTITITHKTHALML
Protobothropsmucrosquamatus-ep      S--KMPMQVDGEPWAQGPCTITITHKTHALVL
Serinuscanaria-epsilon              S--KMPMQVDGEPWAQGPCTVTITHKTHALML
Sturnusvulgaris-epsilon             S--KMPMQVDGEPWAQGPCTVTITHKTHALML
Xenopustropicalis-epsilon           S--KMPMQVDGEPWAQGPCTVTITHKTHALML
Pundamilianyererei-epsilon          S--TMPMQVDGEPWAQGPCTITITHKTQAFML
Homosapiens-alpha                   T---LPMQIDGEPWMQTPCTIKITHKNQMPML
Rattusnorvegicus-alpha              T---LPMQVDGEPWMQAPCTIKITHKNQMPML
Pythonbivittatus-alpha              H---LPMQIDGEPWMQAPCTIRITHKNQAPML
Protobothropsmucrosquamatus-al      H---LPMQIDGEPWMQPPCTIRITHKNQAPML
Maylandiazebra-epsilon              S--TMPMQVDGEPWAQGPCTITITHKTQAFML
Sturnusvulgaris-theta               P---IPVQVDGEPWIQAPGQIIISAAGPKVHM
Serinuscanaria-theta                P---IPVQVDGEPWIQAPGQIIISAAGPKVHM
Rattusnorvegicus-theta              A---TPVQVDGEPWIQAPGHMIISATAPKVHM
Homosapiens-theta                   A---TPVQVDGEPWVQAPGHMIISAAGPKVHM
Protobothropsmucrosquamatus-th      P---IPVQVDGEPWIQPPGQIIISAAGPKVHM
Xenopustropicalis-theta             P---IPVQVDGEPWIQPPGQIIISAAGPKVHM
Pundamilianyererei-theta            P---IPVQVDGEPWIQPPGHIIISAAGPKVRM
Maylandiazebra-theta                P---IPVQVDGEPWIQPPGHIIISAAGPKVRM
Rattusnorvegicus-beta               S---LPMQIDGEPWMQTPCTIKITHKNQAPML
Xenopustropicalis-beta              S---LPMQIDGEPWMQTPCTIKITHKNQAPML
Protobothropsmucrosquamatus-be      S---LPMQIDGEPWLQTPCKIKITHKNQAPVL
Serinuscanaria-beta                 S---LPMQIDGEPWMQTPCTIKITHKNQAPML
Pundamilianyererei-beta             S---LPMQIDGEPWMQTPCTIEIVHKNQAPML
Maylandiazebra-beta                 S---LPMQIDGEPWMQTPCTIEIVHKNQAPML
Homosapiens-beta                    S---LPMQIDGEPWMQTPCTIKITHKNQAPML
Pythonbivittatus-delta              DE-GVPVQVDGEAWIQPPGYIWIVHKNRAQTL
Protobothropsmucrosquamatus-de      DE-GVPVQVDGEAWIQPPGYIWIVHKNRAQTL
Sturnusvulgaris-delta               EE-GVPVQVDGEAWIQPPGYIWIVHKNRAQTL
Xenopustropicalis-delta             DE-GVPVQVDGEAWIQPPGFIRIVHKNRTQTL
Homosapiens-eta-other               DE-GVPVQVDGEAWVQPPGIIKIVHKNRAQML
Serinuscanaria-delta                EE-GVPVQVDGEAWIQPPGYIWIVHKNRAQTL
Pundamilianyererei-delta            DE-GVPVQVDGEAWIQPPGYIKIIHKNRTQTL
Maylandiazebra-delta                DE-GVPVQVDGEAWIQPPGYIKIIHKNRTQTL
Homosapiens-delta-other             DE-GVPVQVDGEAWVQPPGYIRIVHKNRAQTL
Rattusnorvegicus-delta              DE-GVPVQVDGEAWIQPPGYIRIVHKNRAQTL
```

```
Rattusnorvegicus-eta                DE-GVPVQVDGEAWVQPPGIIKIVHKNRAQML
Maylandiazebra-eta                  DE-GVPIQVDGEAWIQPPGVIKIQHKNRAQML
Serinuscanaria-eta                  DE-GVPVQVDGEAWIQPPGVIKIIHKNRAQML
Sturnusvulgaris-eta                 DE-GVPVQVDGEAWIQPPGVIKIIHKNRAQML
Xenopustropicalis-eta               EE-GVPVQVDGEAWIQPPGVIKIVHKNRAQML
Pythonbivittatus-eta                DE-GVPVQVDGEAWIQPPGIIKIVHKNRAQML
Protobothropsmucrosquamatus-et      DE-GVPVQVDGEAWIQPPGIIKIVHKNRAQML
Serinuscanaria-kappa                DE-GVPVQVDGEAWIQPPGIIKIQHKNRAQML
Sturnusvulgaris-kappa               DE-GVPVQVDGEAWIQPPGIIKIQHKNRAQML
Protobothropsmucrosquamatus-ka      DE-GVPVQVDGEAWIQPPGVIKIQHKNRAQML
Homosapiens-kappa                   EE-GIPVQVDGEAWIQRPGLIKIRYKNAAQML
Bostaurus]Kappa                     EE-GVPVQVDGEAWVQRPGLIKIRYKNTAQML
Rattusnorvegicus]kappa              EG-GIPVQVDGEAWIQKPGLIKIKYKNTAQML
Maylandiazebra-iota                 T---VPVQVDGEPCRLAPSTLRISLRNQANMV
Pundamilianyererei-iota             T---VPVQVDGEPCRLAPSTLRISLRNQANMV
Homosapiens-iota                    S---IPMQVDGEPCRLAPAMIRISLRNQANMV
Rattusnorvegicus-iota               S---IPMQVDGEPCRLAPAMIRISLRNQANMV
Sturnusvulgaris-iota                S---IPMQVDGEPCRLAPSLIRISLRNQANMV
Serinuscanaria-iota                 S---IPMQVDGEPCRLAPSLIRISLRNQANMV
Protobothropsmucrosquamatus-io      S---IPMQVDGEPCRLAPSLIRISLRNQANMV
Homosapiens-zeta                    A---IPVQVDGEPCKLAASRIRIALRNQATMV
                                         *:*:***.       : *        :
```
