# Supplementary material for: Phylogenetic analysis of the diacylglycerol kinase family of proteins and identification of multiple highly-specific conserved inserts and deletions within the catalytic domain that are distinctive characteristics of different classes of DGK homologs
Source: PLoS One. 2017 Aug 22;12(8):e0182758. doi: 10.1371/journal.pone.0182758 (PMC5567653; doi:10.1371/journal.pone.0182758)
Supplement: S1 Table — (PDF) [file pone.0182758.s002.pdf]

S1 Table. Sequence information for different DGK homologs used in phylogenetic studies

Species name	DGK Isozyme	Accession number	Length (amino acids)
Homo sapiens	gamma	NP_001337.2	791
Rattus norvegicus	gamma	NP_037258.1	788
Serinus canaria	gamma	XP_009087565.1	781
Sturnus vulgaris	gamma	XP_014725873.1	904
Python bivittatus	gamma	XP_015743514.1	966
Protobothrops mucrosquamatus	gamma	XP_015669628.1	906
Homo sapiens	beta	XP_016867279.1	725
Rattus norvegicus	beta	NP_062177.1	801
Protobothrops mucrosquamatus	beta	XP_015672258.1	801
Serinus canaria	beta	XP_009084260.1	802
Xenopus tropicalis	beta	NP_001096214.1	785
Pundamilia nyererei	beta	XP_013767883.1	783
Maylandia zebra	beta	XP_004560243.1	783
Homo sapiens	alpha	XP_005268745.1	773
Rattus norvegicus	alpha	XP_008763241.1	763
Python bivittatus	alpha	XP_007427065.1	744
Xenopus tropicalis	alpha	XP_002934577.1	744
Protobothrops mucrosquamatus	alpha	XP_015671806.1	744
Maylandia zebra	alpha	XP_004560486.1	731
Homo sapiens	epsilon	NP_003638	567
Rattus norvegicus	epsilon	NP_001034430.1	407
Python bivittatus	epsilon	XP_007428710.1	570
Serinus canaria	epsilon	XP_009092615.1	554
Protobothrops mucrosquamatus	epsilon	XP_015672712.1	569
Sturnus vulgaris	epsilon	XP_014733739.1	554
Xenopus tropicalis	epsilon	XP_002937855.1	554
Pundamilia nyererei	epsilon	XP_005737401.1	576
Maylandia zebra	epsilon	XP_004538850.1	576
Sturnus vulgaris	delta	XP_014735016.1	954
Homo sapiens	delta	XP_011510341.1	968
Serinus canaria	delta	XP_009087492.1	1160
Rattus norvegicus	delta	XP_006226999.1	1169
Xenopus tropicalis	delta	XP_012826605.1	1148
Python bivittatus	delta	XP_015745911.1	1128
Protobothrops mucrosquamatus	delta	XP_015669192.1	1289
Pundamilia nyererei	delta	XP_013770987.1	1096
Maylandia zebra	delta	XP_012780570.1	1096
Homo sapiens	kappa	XP_016884757.1	1242
Rattus norvegicus	kappa	XP_008771334.1	1065
Serinus canaria	kappa	XP_009088490.1	1086
Sturnus vulgaris	kappa	XP_014746482.1	1149
Protobothrops	kappa	XP_015687893.1	1184

mucrosquamatus			
Bos taurus	kappa	NP_001030359.2	1215
Homo sapiens	eta	NP_001284358.1	919
Maylandia zebra	eta	XP_004573039.1	1088
Python bivittatus	eta	XP_007425896.1	1173
Protobothrops mucrosquamatus	eta	XP_015666543.1	1186
Serinus canaria	eta	XP_009101226.1	924
Sturnus vulgaris	eta	XP_014746947.1	995
Xenopus tropicalis	eta	XP_012813202.1	1190
Rattus norvegicus	eta	XP_006222097.1	1077
Homo sapiens	iota	XP_016868274.1	879
Rattus norvegicus	iota	NP_942077.2	1050
Protobothrops mucrosquamatus	iota	XP_015678872.1	1064
Maylandia zebra	iota	XP_004573783.1	965
Pundamilia nyererei	iota	XP_005747329.1	944
Sturnus vulgaris	iota	XP_014737082.1	867
Serinus canaria	iota	XP_009092397.1	868
Homo sapiens	zeta	NP_001186196.1	928
Serinus canaria	zeta	XP_009100702.1	1003
Sturnus vulgaris	zeta	XP_014737418.1	883
Protobothrops mucrosquamatus	zeta	XP_015672706.1	1192
Rattus norvegicus	zeta	NP_112405.1	929
Python bivittatus	zeta	XP_007429871.1	1177
Xenopus tropicalis	zeta	XP_002934142.2	970
Maylandia zebra	zeta	XP_014263169.1	1033
Pundamilia nyererei	zeta	XP_013770097.1	1310
Homo sapiens	theta	NP_001338.2	942
Rattus norvegicus	theta	NP_001185733.1	937
Xenopus tropicalis	theta	XP_012808691.1	847
Serinus canaria	theta	XP_009094351.1	902
Protobothrops mucrosquamatus	theta	XP_015676370.1	958
Sturnus vulgaris	theta	XP_014743606.1	919
Pundamilia nyererei	theta	XP_005737796.1	919
Maylandia zebra	theta	XP_004552088.1	920
